# Overexpression of *PtDXS* Enhances Stress Resistance in Poplars

**DOI:** 10.3390/ijms20071669

**Published:** 2019-04-03

**Authors:** Hui Wei, Ali Movahedi, Chen Xu, Weibo Sun, Amir Almasi Zadeh Yaghuti, Pu Wang, Dawei Li, Qiang Zhuge

**Affiliations:** 1 Co-Innovation Center for Sustainable Forestry in Southern China, Key Laboratory of Forest Genetics & Biotechnology, Ministry of Education, College of Biology and the Environment, Nanjing Forestry University, Nanjing 210037, China; 15850682752@163.com (H.W.); ali_movahedi@njfu.edu.cn (A.M.); xuchenidea@hotmail.com (C.X.); czswb@njfu.edu.cn (W.S.); Amir_20364@yahoo.com (A.A.Z.Y.); 18705155218@163.com (P.W.); dwli@njfu.edu.cn (D.L.); 2 Jiangsu Provincial Key Construction Laboratory of Special Biomass Resource Utilization, Nanjing Xiaozhuang University, Nanjing 211171, China

**Keywords:** *Populus trichocarpa*, DXS, elicitor treatments, ABA, GA

## Abstract

1-Deoxy-d-xylulose-5-phosphate synthase (DXS) is the rate-limiting enzyme in the plastidial methylerythritol phosphate pathway (MEP). In this study, *PtDXS* (XM_024607716.1) was isolated from *Populus trichocarpa*. A bioinformatics analysis revealed that *PtDXS* had high homology with the *DXS*s of other plant species. *PtDXS* expression differed among plant tissues and was highest in young leaves and lowest in roots. The recombinant protein was produced in *Escherichia coli* BL21 (DE3), purified, and its activity evaluated. The purified protein was capable of catalyzing the formation of 1-deoxy-d-xylulose-5-phosphate (DXP) from glyceraldehyde-3-phosphate and pyruvate. A functional color assay in *E*. *coli* harboring pAC-BETA indicated that *PtDXS* encodes a functional protein involved in the biosynthesis of isoprenoid precursors. The treatment of *P*. *trichocarpa* seedlings with 200 μM abscisic acid (ABA), 200 mM NaCl, 10% polyethylene glycol_6000_, and 2 mM H_2_O_2_ resulted in increased expression of *PtDXS*. The ABA and gibberellic acid contents of the transgenic lines (Poplar Nanlin 895) were higher than wild types, suggesting that DXS is important in terpenoid biosynthesis. Overexpression of *PtDXS* enhanced resistance to *S*. *populiperda* infection. Furthermore, the transgenic lines showed decreased feeding by *Micromelalopha troglodyta*, supporting the notion that PtDXS is a key enzyme in terpenoid biosynthesis.

## 1. Introduction

Higher plants have two distinct isoprenoid biosynthesis pathways, the chloroplastic methylerythritolphosphate (MEP) pathway, which is responsible for producing monoterpenoids and diterpenoids, and the cytosolic mevalonic acid (MVA) pathway, which is responsible for synthesizing sesquiterpenoids and triterpenes [1,2,3]. Both the MEP and MVA pathways provide isopentenyl diphosphate (IPP) and dimethylallyl diphosphate (DMAPP) for isoprenoid biosynthesis [4,5]. All known isoprenoids are necessary for life [6]. For example, the isoprenoid vitamin A plays a role in human growth and development as well as in immune system maintenance. Isoprenoids are also implicated in defense against biotic and abiotic stresses, intracellular signal transduction and vesicular transport, and formation of cellular and organelle membranes [7,8,9,10,11].

In the first important step in the MEP pathway, 1-deoxy-d-xylulose-5-phosphate synthase (DXS) catalyzes the condensation of pyruvate and glyceraldehyde-3-phosphate (G-3-P) to form 1-deoxy-d-xylulose 5-phosphate (DXP) [12]. DXP synthesis requires Mg^2+^ or Mn^2+^ and thiamine pyrophosphate (TPP) as a cofactor [13,14]. Next, 1-deoxy-d-xylulose 5-phosphate reductoisomerase (DXR) catalyzes the conversion of 1-deoxy-D-xylulose 5-phosphate to 2-C-methyl-d-erythritol-4-phosphate (MEP), which requires NADPH as a cofactor as well as Mg^2+^ or Mn^2+^ [15].

*DXS* genes from *Ginkgo biloba*, *Aquilaria sinensis*, *Arabidopsis thaliana*, conifers, maize, and soybean have been cloned and characterized [16,17,18,19]. Transformation of tomato plants with bacterial *DXS*s resulted in significant increases in carotenoid content [20]. In *DXS*-overexpressing *A*. *thaliana*, the contents of terpenoids–tocopherols, chlorophyll, carotenoids, and abscisic acid (ABA) were significantly higher than wild types (WT) [21]. In addition, the contents of these terpenoids in *DXS*-underexpressing *A*. *thaliana* were significantly lower than WT plants. The chlorophyll content of *DXS*–over- and under-expressing *A*. *thaliana* was 130% to 142% and 65% to 84% that of the WT, respectively. In addition, the hypocotyls of *DXS*–over- and under-expressing *A*. *thaliana* were significantly shorter and longer than WT plants, respectively. *AtDXS* from *A*. *thaliana* was transferred into lavender, resulting in a significantly higher essential oil content in T0 and T1 transgenic seedlings than in WT plants, but the carotenoid and chlorophyll contents were unaffected [22]. Transfer of a bacterial *DXS* gene into potato resulted in tuber lengthening and premature flowering, indicating DXS is involved in phenotypic regulation. In addition, the carotene content increased two-fold and that of lycopene by six- to seven-fold compared to the control [21]. Thus, DXS is the rate-limiting enzyme in isoprenoid synthesis and a variety of physiological processes.

In this work, we report the cloning and characterization of *DXS* from *Populus trichocarpa*. We also evaluated *PtDXS* expression in different plant tissues and in the presence of abiotic stresses. Overexpression of *PtDXS* enhanced the tolerance of poplar to biotic stresses.

## 2. Results

### 2.1. Molecular Cloning and Sequence Analysis of PtDXS

A 2900 bp full-length cDNA, named *PtDXS* (GenBank accession no. XM_024607716.1) was isolated from leaves of *P*. *trichocarpa*. It contained a 2166 bp ORF encoding a peptide of 721 amino acids, a 351 bp 5′-untranslated region, and a 383 bp 3′-untranslated region (Figure S1). The PtDXS protein had a predicted molecular mass of 77.72 kDa and an isoelectric point of 6.62 (http://web.expasy.org/cgi-bin/protparam/protparam). The deduced amino acid sequence of *PtDXS* was similar to those of *DXS*s from other species; e.g., *Alpinia officinarum* (AEK69518.1, 80.72% identity), *Hevea brasiliensis* (ABD92702.1 86.78% identity), *Ricinus communis* (XP_015573388.1, 86.17% identity), and *Theobroma cacao* (XP_017975597.1, 86.56% identity) (Figure S2).

Plant DXS proteins contain a chloroplast transit peptide, which is consistent with the subcellular localization of the MEP pathway [23]. A predicted chloroplast transit peptide with a conserved VXA cleavage site [24] was present at the N-terminus of PtDXS and was rich in hydroxylated serine and threonine residues and lacked acidic amino acids such as aspartic acid and glutamic acid (Figure S2). Thiamin pyrophosphate (TPP) is a cofactor of DXS and is indispensable for its activity [25]. PtDXS also contains an N-terminal TPP-binding domain, which begins and concludes with the highly conserved sequences GDG and LNDN, respectively (Figure S2). A glutamic acid residue, which is thought to be related to transketolase activity, was found in the middle of PtDXS. Moreover, DRAG and PSD domains, which are considered to be related to pyridine binding, were present at the C-terminus, similar to the DXSs of other plant species (Figure S2).

### 2.2. Structural and Phylogenetic Analyses of PtDXS

**Figure 1:**
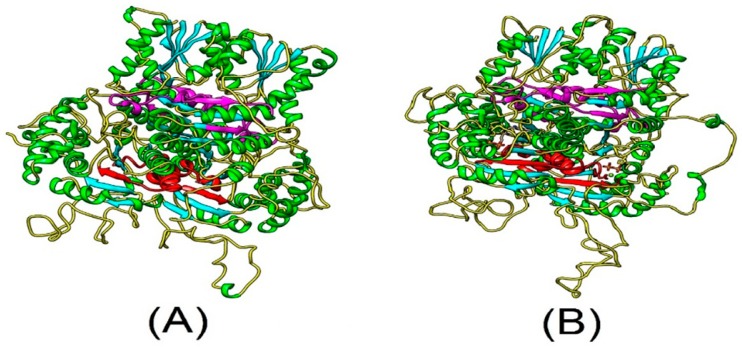
Predicted tertiary structures of AtDXS and PtDXS Green, cyan, and yellow, α-helices, β-strands, and random coils, respectively. Red and magenta, TPP and DRAG domain motifs, respectively. (**A**) Tertiary structure of AtDXS; (**B**) Tertiary structure of PtDXS.

The predicted three-dimensional structures of PtDXS and AtDXS were determined using the SWISS-MODEL server (http://www.expasy.org/swissmod/SWISS-MODEL.html) (Figure 1). PtDXS and AtDXS (NP_193291.1) comprised coils, helices, and strands. These two proteins also contain a TPP-binding domain [26] in the N-terminal region and an NADH-binding domain, which plays an important functional role. Therefore, we speculated PtDXS might have a biological function similar to AtDXS.

**Figure 2:**
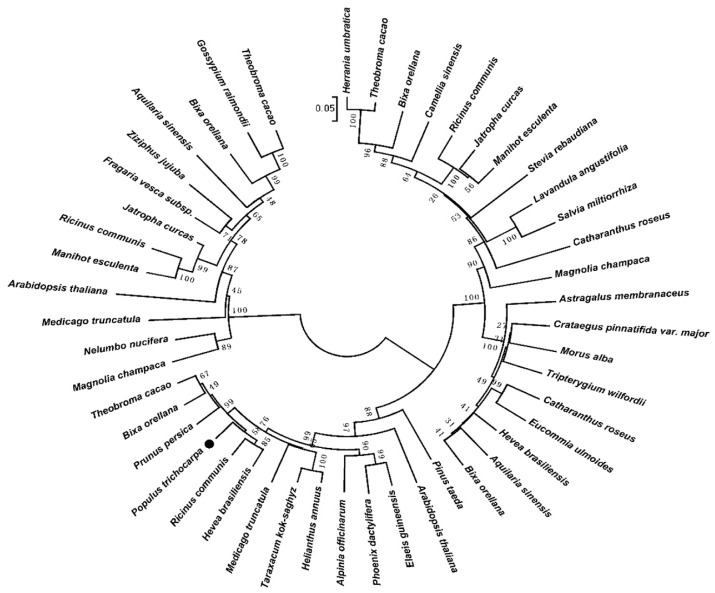
Phylogenetic tree of *Populus trichocarpa.* Amino acid sequences of PtDXS (XP_024463484.1) and other DXS proteins. The tree was constructed using the neighbor-joining method in MEGA 5.1 and was bootstrapped 1000 times. Bootstrap percentages are indicated at the branch points. In all cases, the tree topologies obtained using the neighbor-joining, minimum-evolution, and maximum-parsimony methods were identical. The GenBank accession numbers of the DXS sequences are as follows: *Alpinia officinarum* (AEK69518.1), *Medicago truncatula* (CAD22530.1), *Pinus taeda* (ACJ67021.1), *Catharanthus roseus* (ABI35993.1), *Jatropha curcas* (XP_012076628.1), *Fragaria vesca subsp. Vesca* (XP_011459218.1), *Colwellia psychrerythraea* (KGJ90592.1), *Hevea brasiliensis* (ABD92702.1), *Bixa orellana* (AMJ39459.1), *Elaeis guineensis* (XP_019710182.1), *Helianthus annuus* (OTG31837.1), *Phoenix dactylifera* (XP_008795130.1), *Prunus persica* (XP_007225144.1), *Ricinus communis* (XP_015573388.1), *Taraxacum kok-saghyz* (AMB19705.1), *Theobroma cacao* (XP_017975597.1), *Aquilaria sinensis* (AHI62962.1), *Astragalus membranaceus* (AID51428.1), *Bixa orellana* (AMJ39460.1), *Catharanthus roseus* (CAA09804.2), *Crataegus pinnatifida* var. Major (ALL29183.1), *Eucommia ulmoides* (AFU93069.1), *Hevea brasiliensis* (BAF98289.1), *Morus alba* (ALD62471.1), *Tripterygium wilfordii* (AKP20998.1), *Arabidopsis thaliana* (OAP04569.1), *Bixa orellana* (AMJ39461.1), *Camellia sinensis* (ANB66336.1), *Herrania umbratica* (XP_021276650.1), *Jatropha curcas* (XP_012065282.1), *Lavandula angustifolia* (AGQ04154.1), *Magnolia champaca* (ART66976.1), *Manihot esculenta* (XP_021634514.1), *Ricinus communis* (XP_002533688.1), *Salvia miltiorrhiza* (ACQ66107.1), *Stevia rebaudiana* (ALJ30087.1), *Theobroma cacao* (XP_017981933.1), *Aquilaria sinensis* (AFU75320.1), *Arabidopsis thaliana* (NP_196699.1), *Bixa orellana* (AMJ39462.1), *Gossypium raimondii* (XP_012474408.1), *Jatropha curcas* (XP_012076626.1), *Magnolia champaca* (ART66977.1), *Manihot esculenta* (XP_021597449.1), *Medicago truncatula* (XP_003603440.1), *Nelumbo nucifera* (XP_010254310.1), *Ricinus communis* (XP_002514364.1), *Theobroma cacao* (EOY31423.1), and *Ziziphus jujuba* (XP_015885917.1).

A phylogenetic tree was constructed using the full-length amino acid sequence of DXS proteins (Figure 2). Type-I *DXS* genes are constitutively expressed, mainly in plant green tissues, and produce carotenoid and chlorophyll precursors. Type-II *DXS* genes are present in certain plant tissues [27]. The existence of type-III *DXS* genes has been suggested [28]. Type-I DXSs may play an important role in plant primary metabolism, and type-II DXSs in plant secondary metabolism [29]. Thus, based on the analysis of phylogenetic tree and the transcript profile of PtDXS, we speculate that PtDXS (GenBank accession no. XM_024607716.1) may be a type-I DXS, and that it plays an important role in the basic life processes of plants.

### 2.3. Protein Expression, Purification, and Western Blot Analysis

**Figure 3:**
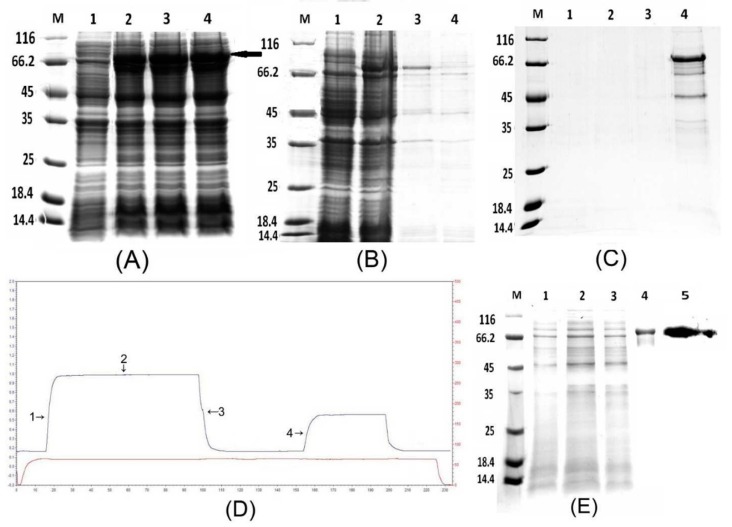
(**A**) Analysis of the fusion protein by 12% SDS-PAGE. Lane M: molecular mass marker; lane 1: negative control; lanes 2–4: colonies 1–3 (induced with 1 mM IPTG). The molecular weight of the target protein was approximately 78.6 kDa (black arrow). (**B**) Analysis of the supernatant and precipitate by SDS-PAGE. Lane M: molecular mass marker; lane 1: negative control; lane 2: colonies induced by 1 mM IPTG; lane 3: precipitate; lane 4: supernatant. (**C**) Denaturation of PtDXS. Lane M: molecular mass marker; lane 1: 2 M urea; lane 2: 4 M urea; lane 3: 6 M urea; and lane 4: 8 M urea. (**D**) Ni-IDA affinity chromatography of the fusion protein. Marks 1 and 2: flow-through; mark 3: wash; and mark 4: elution. (**E**) Purification of PtDXS. Lane M: molecular weight marker; lanes 1, 2: flow-through; lane 3: wash; lane 4: elution; lane 5: western blot analysis of purified PtDXS using a monoclonal antibody against the 6×His tag.

To study the function of PtDXS protein, we firstly needed to obtain PtDXS protein. By using prokaryotic expression and protein purification technology, we successfully expressed and purified PtDXS protein (Figure 3). To identify whether the prokaryotic expression system could express the target protein, the SDS-PAGE analysis was performed. IPTG-induced bacteria showed a specific band of the expected size, 78.6 kDa (including the 6×His tag, Figure 3A). This was consistent with the predicted protein (77.72 kDa). In addition, SDS-PAGE analysis has been carried out to identify whether the expressed PtDXS protein exists in the supernatant or in the precipitation. The result showed that the target protein was detected in the precipitate, suggesting its sequestration in inclusion bodies (Figure 3B). The processes of denaturation and renaturation were used to release inclusion bodies. Inclusion bodies were denatured in 2, 4, 6, and 8 M urea (Figure 3C). Continuously, we obtained the pure target protein PtDXS through the combined techniques of dilution, renaturation phase changes in dialysis, re-dissolution and finally, Ni-IDA resin. To isolate PtDXS fusion protein, we captured His-tagged protein using Ni-IDA resin, washed frequently in buffer containing 20 mM imidazole, and eluted in buffer containing 250 mM imidazole (Figure 3D,E).

Western blotting of PtDXS showed the presence of His-DXS for confirming that the produced peptide in *E*. *coli* BL21 (DE3) was DXS (Figure 3E).

### 2.4. Functional Analysis of PtDXS

To assess the function of PtDXS, preliminary enzymatic assays were conducted using HPLC-MS/MS. A comparison of the retention times and mass fragmentation patterns of samples with those of the DXP standard confirmed that recombinant *PtDXS* catalyzed formation of DXP from G-3-P and pyruvate. By contrast, no peak or mass fragmentation pattern was detected in the negative control (Figure S3A).

To further analyze the biological activity of PtDXS, the color complementation method was used. *E*. *coli* lacks genes related to β-carotene synthesis. However, transgenic *E*. *coli* reportedly produces carotene [30]. To confirm the function of *PtDXS*, single vector pTrc was transformed into *E*. *coli* as the negative control and two pTrc and pAC-BETA vectors were transformed into *E*. *coli* as the positive control. In addition, pTrc-*PtDXS* and pAC-BETA vectors were transformed into *E*. *coli*. Colonies of *E*. *coli* containing pTrc-PtDXS and pAC-BETA (Figure S3Bc) were darker than *E*. *coli* containing the empty vectors pTrc and pAC-BETA (Figure S3Bb). In addition, the co-transformations demonstrated that *PtDXS* encodes the target functional protein. Therefore, PtDXS plays an important role in the synthesis of carotene.

### 2.5. Tissue Specificity of PtDXS Expression

**Figure 4:**
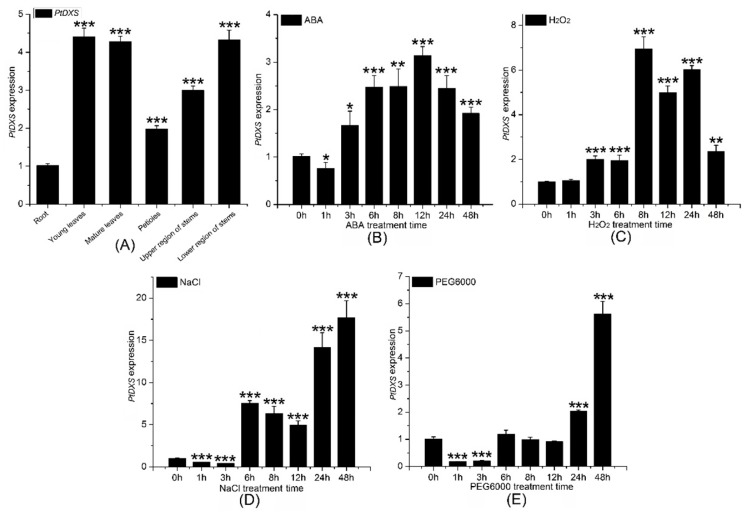
Comparison of *PtDXS* expression in various tissues and in response to stresses. (**A**) Comparison of the *PtDXS* expression in the indicated tissues. Comparison of the *PtDXS* expression level in young leaves followed by different treatments and times (**B**) 200 μM ABA, (**C**) 2 mM H_2_O_2_, (**D**) 200 mM NaCl, and (**E**) 10% PEG6000. All experiments have been normalized by *βactin* as an internal reference; bars represent means ± standard deviation (SD; *n* = 3); three independent biological experiments were performed with three repeats; asterisks represent significant differences in comparing with 0 h treatment as the control (Student’s *t*-test; *, **, and ***, *p* < 0.05, *p* < 0.01, and *p* < 0.001, respectively).

Mature and young leaves, upper and lower stems, petioles, and roots from 2-month-old seedlings of *P. trichocarpa*, which were grown on sterile half-strength Murashige and Skoog medium (pH 5.8), were chosen to extract total RNA. QRT-PCR was used to determine the transcript level of the *PtDXS* gene in different tissues (mature and young leaves, upper and lower stems, petioles, and roots). *PtDXS* was expressed in all tissues examined, and was highest in young leaves and mature leaves, followed by the lower region of the stem, upper region of the stem, petiole, and root. *PtDXS* expression in young leaves was almost 4.6-fold higher than roots (Figure 4A).

### 2.6. Expression Level of PtDXS under Abiotic Stresses

First, 200 mM NaCl, 200 μM abscisic acid (ABA), 10% PEG6000, and 2 mM hydrogen peroxide (H_2_O_2_) were chosen for introducing to 2-month-old seedlings of *P. trichocarpa* grown on sterile half-strength MS medium (pH 5.8). QRT-PCR showed that the expression of *PtDXS* was upregulated by 200 mM NaCl, 200 μM ABA, 2 mM H_2_O_2_, and 10% PEG6000. Treatment with 200 μM ABA significantly upregulated the expression level of *PtDXS* from 3 to 48 h, with a peak at 12 h (Figure 4B). Treatment with 2 mM H_2_O_2_ caused to upregulate the *PtDXS* expression significantly after 3 h, which persisted for 48 h with a peak at 8 h (Figure 4C). Treatment with 200 mM NaCl downregulated the expression of *PtDXS* at 1 and 3 h, followed by an increase from 6–48 h with a peak at 48 h (Figure 4D). Treatment with 10% PEG6000 downregulated the expression of *PtDXS* at1 to 2 days, and the expression of *PtDXS* increased subsequently, and reached the maximum on the 7th day. (Figure 4E). These data suggested that PtDXS protein might play a role in the response to abiotic stresses. In addition, *PtDXS* gene significantly revealed higher expression in responding to H_2_O_2_ stress along certain time points 6, 24 and 48 h in comparing with the other stresses and times (Figure S4).

### 2.7. Overexpression of PtDXS in Nanlin895 Poplar and Response of Transgenic Poplars to 100 mM NaCl and 3% PEG6000 Stresses

To analyze the *PtDXS* functional roles in transgenic poplars, we used the Agrobacterium-mediated transformation to overexpress *PtDXS* gene into WT poplars. Twelve independent transgenic poplars were achieved through screening of regenerated kanamycin-resistant poplars (Figure S5). PCR was performed to determine whether T-DNA was inserted into the genome of Nanlin895 poplars, and qRT-PCR and western blot were used to confirm expression of the target gene (Figure S6). When we used the 35S primer as the upstream primer and *PtDXS*-R primer as the downstream prime, the genomic PCR analyses illustrated that the twelve transgenic lines had the expected bands, but the wild-type poplars had no bands according to the analysis of 1% agarose gel electrophoresis (Figure S6A). In PCR reactions, the *PtDXS*-F and *PtDXS*-R primers isolated one longer fragment > 2166 bp in WT poplars, because of introns, whereas expected targeted bands were appeared in the transgenic lines (Figure S6B). In addition, the result of PCR for *npt*II (kanamycin resistance) gene showed that the twelve transgenic lines exhibited expected bands, but the wild-type poplars exhibited no bands (Figure S6C). The result of southern blot revealed that 2–4 copies of *PtDXS* gene were overexpressed in transgenic lines (Figure S6D). 

The twelve transgenic lines also analyzed by qRT-PCR to show the *PtDXS* transcript levels. qRT-PCR result showed that *PtDXS* gene were expressed in Nanlin895 poplars (Figure S6E). Total protein from WT and transgenic plants was extracted and analyzed by 12% SDS-PAGE (Figure S6F). Furthermore, the results of western blot revealed the presence of PtDXS expressed peptides using His-DXS. Western blotting also revealed that the *PtDXS* gene was stably integrated into the genomes of the transgenic lines and led to expression of the DXS protein using the expression system of the poplars (Figure S6G). 

The growth of wild-type poplars was severely inhibited under the 100 mM NaCl and 3% PEG6000 stress treatments, and the leaves were etiolated and browned (Figure S7A). The transgenic poplar seedlings remained green and alive under 100 mM NaCl and 3% PEG6000 stress treatments, whereas the most WT poplar seedlings revealed yellow and could not be alive (Figure S7B), and most transgenic poplars can survive. The result showed that the survival rates of wild-type poplars were lower than transgenic poplars under 100 mM NaCl and 3% PEG6000 treatments (Figure S7C). Therefore, we resulted that the *PtDXS* overexpression may be able to improve the osmotic stress.

### 2.8. Expression Levels of MVA-, MEP-, ABA-, and GA-Related Genes in PtDXS-Overexpressing Plants

**Figure 5:**
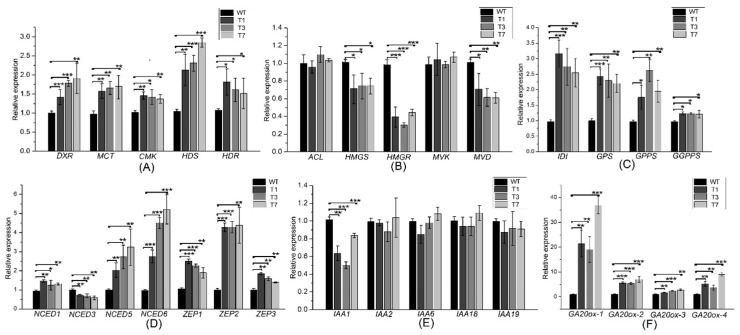
Expression levels of MEP- MVA-, ABA-, IAA-, and GA-related and downstream genes in the transgenic and WT poplar leaves. (**A**) Expression levels of *DXR*, *MCT*, *CMK*, *HDS*, and *HDR*. (**B**) Expression levels of *ACL*, *HMGS*, *HMGR*, *MVK*, and *MVD*. (**C**) Transcript levels of *IDI*, *GPS*, *GPPS*, and *GGPPS*. (**D**) Expression levels of *NCED1*, *NCED3*, *NCED5*, *NCED6*, *ZEP1*, *ZEP2*, and *ZEP3*. (**E**) Expression levels of *IAA1*, *IAA2*, *IAA6*, *IAA18*, and *IAA19*. (**F**) Expression levels of *GA20OX-1*, *GA20OX-2*, *GA20OX-3*, and *GA20OX*-4. All experiments have been normalized using *β-actin* as an internal reference; bars represent means ± SD (*n* = 3). Three independent experiments were performed. The asterisks represent significant differences relative to WT plants (Student’s *t*-test; *, **, and ***, *p* < 0.05, *p* < 0.01, and *p* < 0.001, respectively).

Expression levels of the MEP-related genes *DXR*, *HDS*, *HDR*, *MCT*, and *CMK* were increased significantly in the transgenic plants (Figure 5A). Expression levels of the MVA-related genes *HMGS*, *HMGR*, and *MVD* revealed significant diferences between the transgenic and WT poplars (Figure 5B). In addition, the expression levels of *DXS*, *DXR*, *HDS*, *HDR*, *MVK*, and *MVD* were increased significantly by the overexpression of *PtHMGR* (Figure S8). The expression levels of MVA- and MEP-related genes revealed significant differences between the *PtDXS*- and *PtHMGR*-overexpressing plants. Therefore, we speculated that the MEP pathway might play a leading role in the biosynthesis of terpenoids, and the MVA pathway an auxiliary role. Furthermore, transgenic lines exhibited higher expression levels of the downstream genes *IDI*, *GPS*, *GPPS*, and *GGPPS* in comparing with WT poplars (Figure 5C).

Changes in the expression levels of MVA- and MEP-related genes may affect those of ABA- and GA-related genes. The expression levels of *NCED1*, *NCED5*, *NCED6*, *ZEP1*, *ZEP2*, and *ZEP3* were significantly higher in the transgenic plants, but that of *NCED3* was lower (Figure 5D). The expression levels of *GA20OX-1*, *GA20OX-2*, *GA20OX-3*, and *GA20OX-4* were significantly higher in the transgenic plants (Figure 5F). In addition, while *IAA1* gene revealed significant lower expression in transgenic poplars, *IAA2*, *IAA6*, *IAA18*, and *IAA19* genes revealed no significant differences between transgenic and WT poplars (Figure 5E).

### 2.9. ABA, GA3, and GA4 Contents of PtDXS-Overexpressing Plants

**Figure 6:**
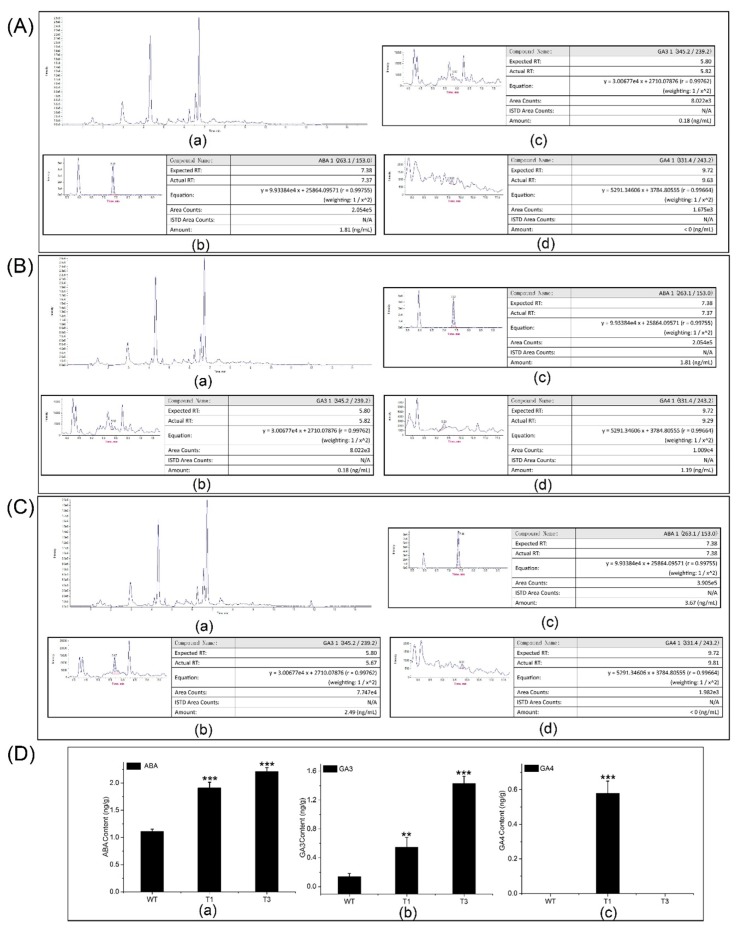
ABA, GA3, and GA4 contents in the transgenic and WT plants by HPLC-MS/MS. (**A**) Raw data and ABA, GA3, and GA4 contents of WT plants. (**B**) Raw data and ABA, GA3, and GA4 contents of T1 plants. (**C**) Raw data and ABA, GA3, and GA4 contents of T3 plants. (**D**) Comparison of the ABA, GA3, and GA4 contents of transgenic and WT plants. Asterisks represent significant differences relative to WT poplars (Student’s *t*-test; **, and ***, *p* < 0.01, and *p* < 0.001, respectively).

HPLC-MS/MS was used to quantify the ABA, GA3, and GA4 contents of the transgenic and WT plants (Figure 6A,D). The ABA content of the transgenic plants (2 ± 0.2 ng/g) was approximately 1.6–2-fold that of the control plants. In addition, there was a significant difference in ABA content between the transgenic and WT plants. The GA3 content of the transgenic plants was approximately 4–14-fold that of the control plants. The highest GA3 content was in the T3 plants (1.51 ng/g). The GA4 content of the T1 plants was 0.65 ± 0.05 ng/g. By contrast, GA4 was not detected in the T3 and WT plants.

### 2.10. Resistance to Septotis Populiperda Infection of PtDXS-Overexpressing Plants and Feeding of Micromelalopha troglodyta on WT and PtDXS-Overexpressing Plants

**Figure 7:**
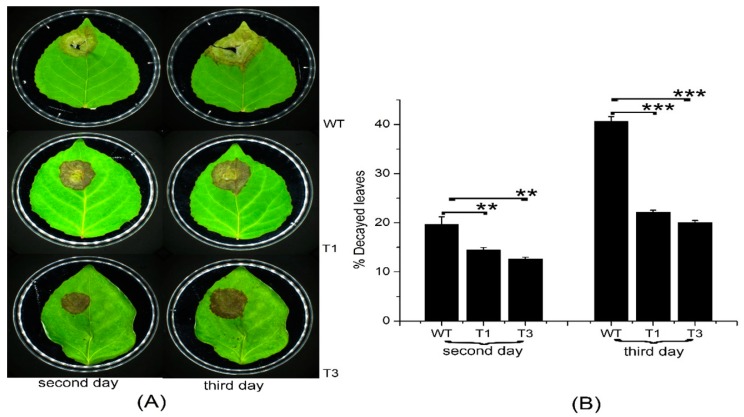
Necrotic symptoms of WT and transgenic plants infected with *S*. *populiperda*. (**A**) Leaves of WT and transgenic plants after inoculation. (**B**) Plaques on WT and transgenic plants. Three independent experiments were performed; three independent experiments were performed; asterisks represent significant differences relative to WT plants (Student’s *t*-test; **, and ***, *p* < 0.01, and *p* < 0.001, respectively).

**Figure 8:**
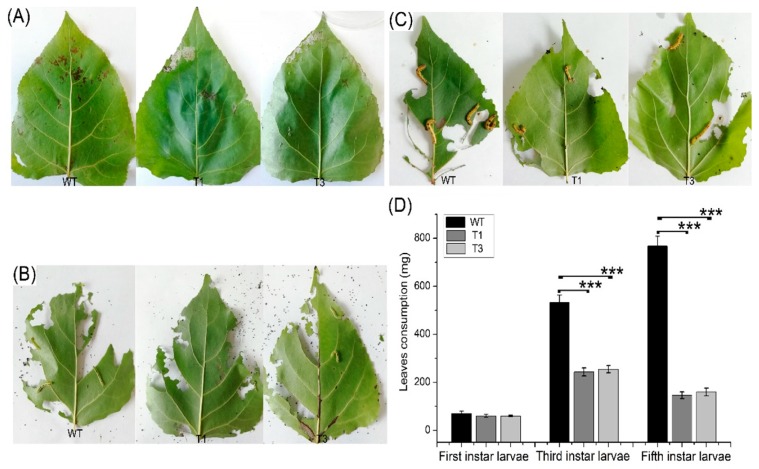
*M*. *troglodyte* feeding assay. (**A**) Leaves of WT and *PtDXS*-transgenic plants after initiating to be eaten by first instar larvae. (**B**) Leaves of WT and *PtDXS*-transgenic plants after after initiating to be eaten by third instar larvae. (**C**) Leaves of WT and *PtDXS*-transgenic plants after after initiating to be eaten by fifth instar larvae. (**D**) Quantitative data for leaves of WT and *PtDXS*-transgenic plants. Three independent experiments were performed; asterisks represent significant differences relative to WT plants (Student’s *t*-test; ***, and *p* < 0.001, respectively).

Most terpenoids have bacteriostatic and bactericidal effects and can enhance plant disease resistance. In addition, terpenoids can resist natural enemies. For example, terpenoids as food inhibitors and toxic substances have a direct effect on insect feeding, as well as a toxic influence on insects during feeding [31]. *Septotis populiperda* causes large-spot disease of poplar. Conidia appeared in WT plants 2 days after inoculation, compared to 4 days in the transgenic plants (Figure 7A). Moreover, the spread of pathogens was greater in the WT than in the transgenic plants (Figure 7B). *Micromelalopha troglodyta* larvae nibble on poplar leaves, which markedly reduces the quality of poplar. Feeding of *Micromelalopha troglodyta* was reduced on the leaves of the transgenic plants (Figure 8A,C). In addition, *Micromelalopha troglodyta* preferred leaves of the WT to those of the transgenic plants (Figure 8A,C). Finally, feeding by *Micromelalopha troglodyta* on WT poplar leaves was greater than that on transgenic plants (Figure 8D).

## 3. Discussion

The enzymes of the MEP pathway produce precursor compounds. DXS is the rate-limiting enzyme in the MEP pathway. We characterized and evaluated the expression of *DXS* from *P*. *trichocarpa*. *PtDXS* encoded a protein with TPP-binding and DRAG domains. The amino acid sequence of PtDXS (XP_024463484) showed a high level of similarity with those of *Alpinia officinarum* (AEK69518.1), *Hevea brasiliensis* (ABD92702.1), *Ricinus communis* (XP_015573388.1), and *Theobroma cacao* (XP_017975597.1). Type-I and -II DXSs may play an important role in plant primary and secondary metabolism, respectively, and type-III DXSs are commonly found in plant genomes, but the functions of their encoded enzymes are unknown [32]. PtDXS is a type-I DXS and plays an important role in basic life processes.

*DXS* expression is related to flower and fruit development, and circadian rhythm. In addition, the expression patterns of *DXS* genes in different families (such as Type-I, -II, and -III *DXS*s) may vary in the same species. The expression level of *DXS* in rose petals increased from the bud to blooming stages. Subsequently, the expression level of *DXS* decreased as flowers decayed. Thus, *DXS* is related to flower development [33]. The circadian clock regulates the expression level of *DXS* in jasmine petal. Tomato *SlDXS2* is highly expressed in flower [34] and *DXS* from *Dendrobium candidum* in the protocorm [35]. In addition, the expression level of *DXS* differs according to the stage of fruit development [36]. *DXS* expression is highest at the early stage of leaf development [37] in plants whose leaves are the major organs of terpenoid synthesis. The expression of *DXS*s in different types of the same species may be the same or different. The expression of *DXS* in *Aquilaria sinensis (Lour.) Spreng* was high in stems, leaves and roots [16]. *DXS1* from *Salvia miltiorrhiza* was expressed in leaves, stems, and roots, and was highest in leaves and lowest in root. However, expression of *DXS2* from *S*. *miltiorrhiza* was highest in root [38]. In this study, *PtDXS* was constitutively expressed in mature and young leaves, upper and lower regions of stems, petioles, and roots of poplar. *PtDXS* expression was highest in young leaves, followed by mature leaves, and was lowest in root; this is in agreement with a previous report [39]. Type-I *DXSs* are expressed in all tissues, except root, of *M*. *truncatula*, *Lycopersicon esculentum*, and *Nicotiana tabacum* [40], which is consistent with our results. *PtDXS* plays an important role in primary metabolism and growth and development. 

Hormones, light, circadian rhythm, developmental degree, and sucrose concentration regulate the expression of *DXS*genes [41]. Treatment of *Dendrobium officinale* with ABA, salicylic acid (SA), jasmonic acid (JA), and brassinosteroids resulted in increased *DXS* expression in the protocorm [35]. In *Aquilaria sinensis*, the expression of *AsDXS1* is regulated by physical and chemical factors, but that of *AsDXS2* and *AsDXS3* is regulated only by physical and chemical factors. These three genes were regulated by methyl jasmonate, but at different times [16]. Treatment with 200 mM NaCl resulted in *PtDXS* expression 18-fold higher than that in untreated plants. Treatment with 200 μM ABA significantly upregulated the expression of *PtDXS* from 3 to 48 h, with a peak at 12 h. Treatment with 2 mM H_2_O_2_ resulted in peak expression of *PtDXS* at 48 h, when it was 18-fold that of the control. The expression of *PtDXS* decreased during the first 2 days of treatment with 10% PEG6000, and increased to a peak at 7 days. Thus, we speculate that *PtDXS* may facilitate development and improvement of poplar resistance to salt and drought stresses. Recombinant PtDXS was capable of catalyzing the formation of DXP from G-3-P and pyruvate. The color complementation method has been used to assess the function of DXS in *Amomum villosum* and *Camptotheca acuminata* [42]. As reported by Cunningham [43], *E*. *coli* strains lacking genes related to carotenoid synthesis do not produce lycopenes. The result of a colorimetric assay indicated that *PtDXS* encodes a functional protein, which increased the accumulation of β-carotene via the MEP pathway. Therefore, *PtDXS* could help to demonstrate an increasing metabolic flux to the synthesis of isoprene compounds according to the color complementation. 

Genes may be co-expressed with other upstream or downstream genes in their metabolic pathways. Upregulation of the expression of potato *DXS* in *A*. *thaliana* increased the expression of downstream *GGPPS* genes, increasing the carotenoid content. In addition, upregulation of phytoene synthase (*PSY*) expression increased the β-carotene content of transgenic plants [44]. In this study, the expression levels of the MEP-related genes *DXR*, *HDS*, *HDR*, *MCT*, and *CMK* as well as downstream genes were increased significantly in the transgenic plants. By contrast, the expression levels of the MVA-related genes *HMGS*, *HMGR*, and *MVD* were decreased. These data suggest that the MEP pathway may play a leading role in terpenoid biosynthesis, while the MVA pathway plays an auxiliary role.

DXS is related to terpenoid synthesis and regulates the generation of downstream products. As the first key enzyme in terpenoid biosynthesis, overexpression or inhibition of DXS can cause changes in downstream metabolites. The carotenoid, chlorophyll, and ABA contents of *Arabidopsis* harboring exogenous *DXS* increased [44,45]. However, the ABA and GA contents of *Arabidopsis* decreased following transfer of *DXS1* from *Solanum tuberosum* [44]. The expression of *DXS* from *Nicotiana tabacum* was positively related to the carotenoid content of fruit, and silencing of *SIDXS2* reduced the β-carotene content [34]. In this study, the transgenic plants had significantly greater ABA and GA contents than the WT. Thus, the GA and ABA contents are correlated with the expression level of *DXS*, and thus DXS plays an important role in their biosynthesis.

Infection of the root of wheat, maize, and rice by mycorrhizal fungi alters the expression levels of *DXS*s [46]. Mycorrhizal fungi infect the roots of *Medicago truncatula*, which increases the concentration of MtDXS2 [23]. Potato late blight is related to the expression level of *DXS*, and a decrease in *DXS* expression results in decreased levels of terpenoids, which are related to disease resistance [44]. In this study, the transgenic plants showed enhanced resistance to *S*. *populiperda* infection as well as reduced feeding by *M*. *troglodyta.*

We cloned full-length *PtDXS*, which is related to isoprenoid biosynthesis, from *P*. *trichocarpa*. We also assessed the expression pattern of *PtDXS* in various tissues and under abiotic stresses and analyzed the expression levels of MEP- and MVA-related genes in the transgenic and WT plants. Overexpression of *PtDXS* increased the ABA and GA contents, and *PtDXS* expression was positively related to resistance to *S*. *populiperda* and negatively related to feeding by *M*. *troglodyta*. Our findings will facilitate further studies of the functions of *PtDXS* gene in *P*. *trichocarpa.*

## 4. Materials and Methods

### 4.1. Plant Materials and Treatments

*P*. *trichocarpa* was sterilized and grown in Murashige and Skoog (MS) medium at 23 °C for 2 months. *P*. *trichocarpa* was also grown in MS medium supplemented with 200 mM NaCl, 200 μM ABA, or 2 mM H_2_O_2_. Leaves were collected after 1, 3, 6, 8, 12, 24, and 48 h. As drought stress, *P*. *trichocarpa* was treated with 10% polyethylene glycol (PEG) 6000 and leaves were collected after 1, 2, 3, 4, 5, 6, and 7 days. Total RNA was extracted from the young and mature leaves, upper and lower regions of stems, roots, and petioles using an RNAprep Pure Plant Kit (Biomiga Company, San Diego, CA, USA) according to the manufacturer’s instructions. The MMLV reverse transcriptase (TaKaRa, Japan) was used to synthesize cDNA according to the manufacturer’s instructions.

### 4.2. Cloning of Full-Length PtDXS and Rapid Amplification of cDNA Ends

*PtDXS* was amplified by PCR using specific primers (Table 1); the PCR system included 2 µL forward and reverse primers, 2.0 µL cDNA as template, 5.0 µL 10× PCR buffer (Mg^2+^ plus), 1 µL 10 mM dNTPs, 0.5 µL rTaq DNA polymerase (Takara, Japan) and the ddH_2_O was to a constant volume up to 50 µL. Also, the PCR reaction was performed as follows: 95 °C for 10 min, 35 cycles of 95 °C for 1.5 min, 58 °C for 1.5 min, and 72 °C for 2 min and, finally, 72 °C for 10 min. In addition, the PCR product was purified according to the manufacturer’s instructions (AXYGEN, Suzhou, China), and the purified product was cloned into the PEASY-T3 vector (TransGen Biotech, Beijing, China) based on the complementary cohesive end. The vector was transformed into *Escherichia coli* TransTI. Positive clones were selected by blue and white spot induced by X-Gal and IPTG and the reconstructed plasmids were sequenced by GenScript Company (Nanjing, China).

Rapid amplification of cDNA ends (RACE) was used to amplify the 3′ and 5′ untranslated regions of *PtDXS*. Next, the 5′ and 3′ fragments were sequenced using specific primers (Table 1). The 5′- and 3′-RACE amplified fragments were inserted into the PEASY-T3 vector and sequenced. The full-length sequence of *PtDXS* was obtained by aligning the obtained sequences.

### 4.3. Construction of the Expression Vector

According to the *PtDXS* sequence and the restriction enzyme sites in the PET-28a vector, forward and reverse primers were designed and synthesized (Table 1). The target gene was amplified by PCR, and the PCR product and vector were cut with *Bam*HI and *Xho*I. Subsequently, the fusion plasmid was generated using the T4 ligation technique. Subsequently, the recombinant plasmid PET-28a-*PtDXS* was transformed into *E*. *coli* Top10, we can achieve the positive clones according to screening of solid Luria-Bertani broth (LB) medium containing 50 µg/mL kanamycin at 37 °C for 12 h. Moreover, the recombinant plasmid PET-28a-*PtDXS* was extracted by the plasmid extraction kit (AXYGEN) based on the manufacturer’s instructions.

### 4.4. Production and Purification of the Target Protein

We used electroporation to transform the recombinant plasmid PET-28a-*PtDXS* (with no mutation) into *E*. *coli* BL21 (DE3) cells. Next, recombinant *E*. *coli* BL21 (DE3) was induced with 1 mM isopropyl β-d-1-thiogalactopyranoside (IPTG) at 220 rpm for 4 h at 37 °C. Non-induced culture medium, induced culture medium, supernatant, and sediment were analyzed by 12% sodium dodecyl sulfate-polyacrylamide gel electrophoresis (SDS-PAGE).

The target protein was present in inclusion bodies according to analysis of the supernatant and sediment. Inclusion body denaturation and renaturation were performed as follows: The precipitate was suspended in 20 mL of lysis buffer (20 mM Tris-HCl containing 1 mM phenylmethylsulfonyl fluoride and protease inhibitor cocktail; pH 8.0), sonicated (400 W for 4 s, followed by at 8 s intervals for 20 min), centrifuged at 10,000 r/min for 20 min, and the precipitate collected. The inclusion bodies were washed three times with inclusion body detergent (20 mM Tris, 1 mM ethylenediaminetetraacetic acid, 2 M urea, 1 M NaCl, 1% Triton X-100; pH 8.0). The inclusion bodies were dissolved in solution buffer (20 mM Tris, 5 mM dithiothreitol, 8 M urea; pH 8.0), placed overnight at 4 °C, and centrifuged at room temperature for 15 min at 10,000 r/min. The resulting solution was dropped into buffer (20 mM Tris-HCl, 0.15 M NaCl; pH 8.0), and stirred slowly, and transferred to dialysis bags for dialysis overnight. Using a low-pressure chromatography system, the solution was loaded at a flow rate of 0.5 mL/min onto a Ni-IDA-Sepharose CL-6B affinity chromatography column. The column was rinsed with binding buffer (20 mM Tris-HCl, 10 mM imidazole, 0.15 M NaCl; pH 8.0) and washing buffer (20 mM Tris-HCl, 50 mM imidazole, 0.15 M NaCl; pH 8.0), and elution buffer (20 mM Tris-HCl, 250 mM imidazole, 0.15 M NaCl, pH 8.0) was used to elute the target protein at a flow rate of 1 mL/min. The collected solution was transferred to a dialysis bag and dialyzed overnight against 20 mM Tris-HCl, 0.15 M NaCl (pH 8.0). Purification was verified by 12% SDS-PAGE and western blotting for the 6×His tag.

### 4.5. Detection of 1-Deoxy-d-Xylulose-5-Phosphate In Vitro

PtDXS activity was assayed by detecting DXP in vitro. One milliliter of a reaction mixture containing 110 mM fructose-1,6-diphosphate (pH 7.5), 10 mM MgCl_2_, 120 mM Tris-HCl (pH 7.5), 60 U aldolase, 60 U triose phosphate isomerase, and 5 mM β-mercaptoethanol was heated in a water bath at 25 °C for 1 h. Next, 110 mM sodium pyruvate, 2 mM thiamin pyrophosphate (TPP), and 100 μg of DXS were added to the reaction mixture. The solution was heated in a water bath at 37 °C for 16 h, and concentrated using a 10 kDa filter at 11,000 rpm for 2 min. The solution was finally freeze-dried to powder. *E*. *coli* BL21 (DE3) carrying an empty vector was included as the negative control. In addition, quantitative analysis of PtDXS reaction product was performed by high-performance liquid chromatography (HPLC)/mass spectrometry (MS) using the Agilent poroshell 120 SB-C18 reversed-phase column. The column temperature was set to 30 °C. The mobile phase contained 80% methanol and 20% water (0.1% formic acid) and was eluted using a gradient of 200 μL/min. The MS conditions were as follows: spray voltage of 4000 V (+)/3500 V (−), air curtain of 15 psi, atomizing gas pressure of 45 psi, auxiliary pressure of 60 psi, and atomization temperature of 340 °C. 

### 4.6. Functional Analysis of PtDXS in Escherichia coli

The plasmids pAC-BETA and pTrc-AtIPI provided by Francis X. Cunningham Lab. (Addgene Company, Watertown, MA, USA) were used to investigate the biological function of PtDXS. GGPP synthase (*crtE*), octahydrolycopene synthase (*crtB*), and octahydrolycopene desaturase (*crtI*), all of which are necessary for the synthesis of β-carotene, are present in pAC-BETA. Based on the *PtDXS* sequence and the restriction enzyme sites in the pTrc-AtIPI vector, forward and reverse primers were designed and synthesized (Table 1). The *PtDXS* gene was amplified by PCR, and *PtDXS* was cloned into pTrc-AtIPI by digestion with *Bgl*II and *Not*I and T4 ligation. The vectors pTrc-*PtDXS* and pAC-BETA were co-transformed into *E*. *coli* DH5α, and the co-transformants were screened on solid LB mediums containing 100 µg/mL ampicillin and 50 µg/mL chloramphenicol at 37 °C for 48 h. The two control groups were *E*. *coli* DH5α containing pTrc and pAC-BETA and *E*. *coli* DH5α containing pTrc.

### 4.7. Determination of PtDXS Expression

Movahedi et al. [47] proved that *βactin* (accession number: XM-006370951.1) could be stably expressed in leaves, stems, and roots. Thus, we decided to use *βactin* as an internal reference gene to normalize qPCR for assessing *PtDXS* expression. In addition, we performed RT-PCR to exhibit whether the *βactin* gene could be stably expressed in all tissues and experimental conditions (Figure S9). The PCR reaction was carried out following 95 °C for 7min, (95 °C for 30 s, 58 °C for 30 s, and 72 °C for 30 s)×26, and finally, 72 °C for 10 min. 

The expression level of *PtDXS* was measured by real-time quantitative polymerase chain reaction (qPCR) using total RNA from mature and young leaves, upper and lower regions of stems, petioles, and roots. The primers q-Actin-F and q-Actin-R were used to amplify a 152 bp fragment of *P*. *trichocarpa actin* (Table 1). The StepOnePlus™ Real-Time PCR System (Applied Biosystems, ThermoFisher Company, Waltham, MA, USA) and SYBR Green Master reagents (Roche, F. Hoffmann-La Roche AG Company, Basel, Switzerland) were chosen to perform the quantitative PCR analysis. The real-time quantitative polymerase chain reaction system contained 1 µL each primer, 2 µL cDNA, 10 µL SYBR Green mixture, and 6 µL ddH_2_O. The real-time quantitative polymerase chain reaction was performed as follows: 95 °C for 5 min, 40 cycles at 95 °C for 15 s and 60 °C for 60 s. Triplicate measurements were performed for each parameter, and each data point represents a mean value.

*P*. *trichocarpa* seedlings were subjected to the following stress treatments: 200 mM NaCl, 200 μM ABA, and 2 mM H_2_O_2_ for 0, 1, 3, 6, 8, 12, 24, or 48 h, and 10% PEG6000 for 0, 1, 2, 3, 4, 5, 6, or 7 days. The leaves were collected, and RNA was extracted using a RNeasy Plant Mini Kit (Biomiga Company, San Diego, CA, USA). Subsequently, real-time quantitative polymerase chain reaction (qPCR) was chosen to identify the transcription level of *PtDXS* gene, the *PtActin* gene was used as internal reference. The StepOnePlus™ Real-Time PCR System (Applied Biosystems) and SYBR Green Master reagents (Roche) were chosen to perform the quantitative PCR analysis. The real-time quantitative polymerase chain reaction was performed as above description. The treatments were repeated three times for analyses of *PtDXS* expression in response to abiotic stress treatment.

### 4.8. Overexpression Plasmid Construction and Transformation

The open reading frame (ORF) of *PtDXS* was cloned into pGWB9 using gateway technology. First, the ORF of *PtDXS* was cloned into the pDONRTM/Zeo entry vector using the BP clonase according to the manufacturer’s instructions (Invitrogen, Carlsbad, CA, USA). The method of electroporation was used to transform the recombinant plasmid pDONRTM/Zeo-PtDXS into *E. coli* Top10 cells and the positive clones were selected by solid LB medium containing 30 µg/mL zeocin. Second, using the LR clonase (Invitrogen), recombinant pDONRTM/Zeo-*PtDXS* was recombined with the pGWB9 vector, and the electroporation was used to transform the recombinant plasmid pGWB9-PtDXS into *E. coli* DH5α cells and the positive clones were selected by solid LB medium containing 50 µg/mL Kanamycin. pGWB9-*PtDXS* was introduced into *Agrobacterium* EHA105, which was used to infect poplar leaves. 

Transgenic operations are carried out in the following steps: Resistant shoots were obtained on selective differentiation medium containing 200 mg/L cefotaxime and 50 mg/L kanamycin. Next, putative resistant shoots were cultured on selective shooting MS medium containing 200 mg/L cefotaxime and 25 mg/L kanamycin. Subsequently, the putative resistant shoots were cultured on half-strength MS medium containing 200 mg/L cefotaxime and 15 mg/L kanamycin. Finally, the plants were transferred to the greenhouse. 

To detect whether the *PtDXS* gene was inserted into the poplar genome, PCR reaction was performed using 35S primer and PtDXS-R primer as the upstream primer and the downstream primer as well as the genomic DNA of wild-type and potential transgenic lines as templates, respectively. Also, the PtDXS-F primer and PtDXS-R primer as well as the genomic DNA of wild-type and potential transgenic lines as templates were used to carry out PCR reaction. The *kanamycin* gene was indetified by PCR in twelve transgenic lines and the wild-type poplars. The copy number of transgenic lines was investigated by Southern blotting. The CTAB method was used to achieve 10 μg of genomic DNA, then the genomic DNA was digested with EcoRI at 37 °C for 4 h. Subsequently, the digested genomic DNA was separated on a 0.8% agarose gel at 15 V, and was transferred to a Hybond N+nylon membrane. Finally, based on the manufacturer’s instructions, blotting was performed (Roche, Basel, Switzerland). We perform the PCR to synthesize a digoxygenin (dig)-labeled *Kan*-tagged cDNA fragment (665 bp), and choose Kan-tagged cDNA fragment as a probe for Southern blotting.

In addition, in order to identify whether the *PtDXS* gene was expressed in the poplar genome, twelve transgenic lines were selected for real-time quantitative polymerase chain reaction (qPCR) analysis. Moreover, to identify whether the *PtDXS* gene was translated in poplar, total proteins from both WT and transgenic poplars were extracted and analyzed by 12% SDS-PAGE. Furthermore, the *PtDXS* gene linked to the PGWB9 vector by homologous recombination will be expressed in fusion with the 6×His tag located on the PGWB9 vector. So, western blotting was used to determine whether PtDXS could be specifically recognized by rabbit antiserum against His-DXS expressed in transgenic poplars.

After 1 month of subculture, shoot tips (3 cm high) were cut from wild-type and transgenic poplars and placed on medium containing 3% (*w*/*v*) PEG6000 or 100 mM NaCl under a 16 h light/8 h dark cycle at 23 °C. The survival rate was investigated after 1 week.

### 4.9. Expression Levels of MVA-, MEP-, ABA-, GA-, and IAA-Related Genes

Total RNA was isolated from the leaves of WT and transgenic poplars and the expression levels of MVA-, MEP-, ABA-, and GA-related genes were assessed by real-time quantitative polymerase chain reaction (qPCR) under the conditions. Furthermore, the expression levels of IAA-related genes in the transgenic and WT plants were determined. We choose *PtActin* as the reference gene, and the primers of MVA-, MEP-, ABA-, GA-, and IAA-related genes were shown in Table 1. The StepOnePlus™ Real-Time PCR System (Applied Biosystems) and SYBR Green Master reagents (Roche) were chosen to perform the quantitative PCR analysis. The real-time quantitative polymerase chain reaction was performed as above description. Triplicate measurements were performed for each parameter, and each data point represents a mean value. 

### 4.10. Determination of ABA, GA3, and GA4 Contents

We used the AB Qtrap6500 mass spectrometer and Agilent 1290 high-performance liquid chromatograph with electrospray ionization (ESI-HPLC) to determine the ABA, GA3, and GA4 contents of the leaves of transgenic and WT plants. Hormones were extracted according to the manufacturer’s instructions (Jiancheng Biotechnique, Nanjing, China). Subsequently, we used nitrogen to dry the collected hormone solutions and use 400 μL methanol containing 0.1% formic acid to dissolved samples processed in the previous step. Finally, the collected solution was filtered through a 0.22-μm membrane and detected using HPLC-MS/MS. 

We used methanol containing 0.1% formic acid as the solvent to prepare standard solutions, and the concentrations of ABA, GA3, and GA4 were 0.1, 0.2, 0.5, 2, 5, 20, 50, and 200 ng/mL, respectively. The liquid phase conditions were as follows: a Poroshell 120 SB-C18 column (2.1 × 150, 2.7 m) was used in this study at a column temperature of 30 °C. The mobile phase included A:B = (methanol/0.1% formic acid):(water/0.1% formic acid). Elution gradient: 0–1 min, A = 20%; 1–9 min, A = 80%; 9–10 min, A = 80%; 10–10.1 min, A = 20%; 10.1–15 min, A = 20%. The injection volume was 2 μL. MS conditions were as follows: air curtain gas, 15 psi; spray voltage, 4500 v; atomization pressure, 65 psi; auxiliary pressure, 70 psi; atomization temperature, 400 °C.

### 4.11. Disease-Response and Antifeedant Assays

*S*. *populiperda* was grown at 23 °C on potato dextrose agar (PDA) for 1 week [48]. The same positions of 2-months-old transgenic and WT poplar leaves, grown in a greenhouse, were punctured using a 5 mL syringe needle and inoculated with a small amount of PDA containing the pathogen or the same amount of PDA only (negative control). Infected leaves were counted, and soft-rot symptoms were evaluated periodically. Each experiment was repeated at least three times.

For the *M. troglodyta* feeding assay, we dipped the eggs of *M. troglodyta* in a brush and attached them to the back of the clean poplar leaves, then placed them in flask followed by incubation for cultivation. The temperature of incubator was 25–28 °C with one photoperiod as light/dark = 14h/10h. The same position of 2-years-old transgenic and WT poplar leaves were used to breed first-instar larvae, third-instar larvae, and fifth-instar larvae. The specific feeding process was carried out as follow: Leaves were collected, washed and dried. In addition, the poplar leaves were placed in flasks. Then, first-instar larvae, third-instar larvae, fifth-instar larvae were fed with transgenic and WT leaves separately, and the leaves were replaced once every 2 days. The fresh weight of the remaining leaves and the weight of the larvae were determined at least three times.

## Figures and Tables

**Table 1:** The primers used in this study.

Primer	Direction	Nucleotide Sequence(5′–3′)	Primer	Direction	Nucleotide Sequence(5′–3′)
*PtDXS*	Forward	ATGGCTCTCTCTGCATTTTCT	q-IDI	Forward	CGGGTTGTTGGTCATGACTC
*PtDXS*	Reverse	TTATGATGACATAATCTCCAGAGAGTTT	q-IDI	Reverse	CTCTGTGCAGCATTCCTCAC
3′GSP-1-*DXS*	Forward	TTCCTGATAGGTACATTGACCATG	q-GPS	Forward	AAGCTCACTCTGATGGGGAC
3′Outer	Reverse	TACCGTCGTTCCACTAGTGATTT	q-GPS	Reverse	TGATAGCCTCCTTGTGTGGG
3′GSP-2-*DXS*	Forward	CTGGTCTCACACCATCTCAC	q-GPPS	Forward	TGAAACGGAAACTGGCAGTG
3′Inter	Reverse	ACACCAATTTTGCATTCTTACAAC	q-GPPS	Reverse	TACTGCCACCTTATTGCCCA
UPM	Forward	CTAATACGACTCACTATAGGGCAAG CAAGCAGTGGTATCAAACGCAGAGT(long)	q-GGPPS	Forward	CTGACCTTGTACCAGAGCCA
		CTAATACGACTCACTATAGGGC(short)	q-GGPPS	Reverse	GAGCACTCACCCATTTCACC
5′GSP-1	Reverse	CTGATTGATCTTTTTAAAAGATTGGC	q-NCED1	Forward	TAGAAACGGAGCCAACCCAT
NUP	Forward	AAGCAGTGGTATCAACGCAGAGT	q-NCED1	Reverse	GCTATGCCCGAATGACCATG
5′GSP-2	Reverse	GAGAGAGACAGAGACAGAGAGGTCT	q-NCED3	Forward	GGCTCCTCGTCATTTTCGTC
q-Pt*DXS*	Forward	GAATGCAAAGCCGTTGAAGC	q-NCED3	Reverse	GGCGTGTTTTGTTTTGGGTG
q-Pt*DXS*	Reverse	GTTTCAATTTTATCAGTGCCAAAA	q-NCED5	Forward	TCCACCGGACTCCATTTTCA
q-Actin	Forward	GCCATCTCTCATCGGAATGGAA	q-NCED5	Reverse	TGTCTGTCTTCCAAGCCGAT
q-Actin	Reverse	AGGGCAGTGATTTCCTTGCTCA	q-NCED6	Forward	GAGACTGACGAGGTGACCAA
PET-*PtDXS*	Forward	CGGGATCCATGGCTCTCTCTGCATTT	q-NCED6	Reverse	CACCAATTCTGACCTCCCCT
PET-*PtDXS*	Reverse	CCGCTCGAGTTATGATGACATAATCT	q-ZEP1	Forward	ACAGTCTCTTTCCCATGCCA
pTrc-PtDXS	Forward	GAATGGCTCTCTCTGCATTT	q-ZEP1	Reverse	CCAGAAGCATGTACAGCACC
pTrc-PtDXS	Reverse	AAGGAAAAAATTATGATGACATAATCT	q-ZEP2	Forward	TGACCTTGCTTGGGGATTCT
pGWB9-PtDXS	Forward	GGGGACAAGTTTGTACAAAAAAGCAGGCTCCATGGCTCTCTCTGCATTT	q-ZEP2	Reverse	TTGCTGCCATTCTTGCCATT
pGWB9-PtDXS	Reverse	GGGGACCACTTTGTACAAGAAAGCTGGGTTTATGATGACATAATCT	q-ZEP3	Forward	GGGATGAGAAGAGGAGGCTC
q-DXR	Forward	TTGAAAAGGGTAGCAGAGTC	q-ZEP3	Reverse	TAACACAGCCAGCGTCCATA
q-DXR	Reverse	TTGTTCTCCCTCTTGCTCAC	q-GA20OX-1	Forward	AGATCCTTTGGCGGTCTCAA
q-HDS	Forward	TCTGTTGCGTTGCGAGTATC	q-GA20OX-1	Reverse	TCCGAGAGCTGCTTACCAAA
q-HDS	Reverse	AAGACTGCCATGGTTTGTCC	q-GA20OX-2	Forward	TAACCACCCCTCATCACCAC
q-HDR	Forward	TAACACCTCCCACCTCCAAG	q-GA20OX-2	Reverse	AGTGGGACTTGGAGTTCAGG
q-HDR	Reverse	TAAGGGCATCTTCGACAACC	q-GA20OX-3	Forward	TACCCTCACTCTTGGCACTG
q-MCT	Forward	ACTGCCAGGAAAGGAGAGAC	q-GA20OX-3	Reverse	CCCTGTTCACCACTGCTCTA
q-MCT	Reverse	ACCAAGTACAGCAGCTCCAT	q-GA20OX-4	Forward	TTCTCCTGTCTCCTCCTCCA
q-CMK	Forward	TTCTCATAAAGCCCCCACAG	q-GA20OX-4	Reverse	GCAGTCCTAACAAGCTCAGC
q-CMK	Reverse	AGCAGGGGGCTCTAAATCAT	q-IAA1	Forward	CTCCTTCGAACTCCCAACCT
q-ACL	Forward	CCTCCACAAATCCCTGAAGA	q-IAA1	Reverse	AATTCGGCGAAACACTGGTC
q-ACL	Reverse	CCCACACCATAACCCTGTTC	q-IAA2	Forward	GCTGAATTGGACCGGTTGTT
q-HMGS	Forward	GTCTGCAATAGCTGGGAAGC	q-IAA2	Reverse	CACGCCCGATGGATTTTCAT
q-HMGS	Reverse	GTGTTCATCGGTAGGCGTTT	q-IAA6	Forward	GTGAACAGGTTGCTGCTTCA
q-HMGR	Forward	CTGGGCCATTGTTGCTTAAT	q-IAA6	Reverse	CGTCCTGACTTAATGGCTGC
q-HMGR	Reverse	TCAACTCAGCAGCCCTTTTT	q-IAA18	Forward	TGTAGGTTGCCGGTTGTTTG
q-MVD	Forward	ATGGGTGAGGATGGTGACTG	q-IAA18	Reverse	GCTTTGTGACTCCTTTGCCA
q-MVD	Reverse	ATTGAGCCACATCCGATCCT	q-IAA19	Forward	AAGACATTCCCGCCTCTTGA
q-MVK	Forward	GCAAACCCTATGGGGAAAAT	q-IAA19	Reverse	TTAGCCCTTCTGATGCCCAA
q-MVK	Reverse	TGCATCAAAACATGGAAGGA			

## Supplementary Materials

Supplementary materials can be found at https://www.mdpi.com/1422-0067/20/7/1669/s1.
