# Supplementary material for: Overexpression of PtDXS Enhances Stress Resistance in Poplars
Source: Int J Mol Sci. 2019 Apr 3;20(7):1669. doi: 10.3390/ijms20071669 (PMC6479640; doi:10.3390/ijms20071669)
Supplement: Supplementary file 1 [file ijms-20-01669-s001.pdf]

Supplemental files

```
   1 ttcgtgatttccaattatgtggctccatggagaaggctcgacacagacactgtaatgctg ctggtaactggtaaagactatatatacatcagccaaaattaccacctcttcacttgcaga
 121 cctctctgtctctgtctctctctctctctctgtgtctcttccaacttccgttattaac agacagccacagaccctcctccctccattatttaactaattcgccactaccaccgaacat
 241 tcattagtggggaatttctctcggtgacaagttcttcttcttcttctacactgtttctgtttcttggttctctgtgtttaagcactcttctttgtttttctcgcatctcctatggctctc
                                                                                                                     M  A  L
 361 tctgcatttttctttgcctgctcatgtcaatagtgtcacaagtttagaccaccttcaaaattatagttttttatcttctcatttcttatggagaacagatctgctgtgccaatcttttaaa
     S  A  F  S  L  P  A  H  V  N  S  V  T  S  L  D  H  L  Q  N  Y  S  F  L  S  S  H  F  L  W  R  T  D  L  L  C  Q  S  F  K
 481 aagatcaatcaggtacatgtcaagaaaaggccaaatggggtttgtgcatcactatcagag agcggagagtttccttcacagaggccaccaacacctctcttggacaccgtaaactatcca
     K  I  N  Q  V  H  V  K  K  R  P  N  G  V  C  A  S  L  S  E  S  G  E  F  P  S  Q  R  P  P  T  P  L  L  D  T  V  N  Y  P
 601 attcatatgaaaaatctatctatcaaggagctaaaacaactagcagaggagctgcggtcc gatgttatcttcaatgtttcgaaaactgggggtcacttaggctctagccttggtgttgtg
     I  H  M  K  N  L  S  I  K  E  L  K  Q  L  A  E  E  L  R  S  D  V  I  F  N  V  S  K  T  G  G  H  L  G  S  S  L  G  V  V
 721 gagctcaccgtggctcttcactatgttttcaatagccctcaagacaagatactgtgggat gttggccatcaggcttaccctcacaagattctaactgggagaagagacaagatgcataca
     E  L  T  V  A  L  H  Y  V  F  N  S  P  Q  D  K  I  L  W  D  V  G  H  Q  A  Y  P  H  K  I  L  T  G  R  R  D  K  M  H  T
 841 ataagacagaccaatggattggctggttttacaaagcgatcggagagtgaatatgattgt tttggcacaggtcacagctctaccaccatttctgctggcttgggaatggctgtggggaga
     I  R  Q  T  N  G  L  A  G  F  T  K  R  S  E  S  E  Y  D  C  F  G  T  G  H  S  S  T  T  I  S  A  G  L  G  M  A  V  G  R
 961 gatctaaaaggaagagcgaacaatgtggttgctgttataggtgatggtgccatgacagca ggacaagcttatgaagctatgaacaatgcagggtacctagactctgatatgatcgttatt
     D  L  K  G  R  A  N  N  V  V  A  V  I  G  D  G  A  M  T  A  G  Q  A  Y  E  A  M  N  N  A  G  Y  L  D  S  D  M  I  V  I
1081 cttaatgacaacaaacaggtttctttaccaactgccaatttagatgggccaataccacct gtcggagccttgagcagtgctctcagtaggctgcaatcaaacaggcctctcagagaacta
     L  N  D  N  K  Q  V  S  L  P  T  A  N  L  D  G  P  I  P  P  V  G  A  L  S  S  A  L  S  R  L  Q  S  N  R  P  L  R  E  L
1201 agagaggttgctaagggagttacaaaacaaattggtggaccgatgcatgaattggcagca aaggttgacgaatatgctcgtggaatgatcagtggttctggatcaaccctctttgaagag
     R  E  V  A  K  G  V  T  K  Q  I  G  G  P  M  H  E  L  A  A  K  V  D  E  Y  A  R  G  M  I  S  G  S  G  S  T  L  F  E  E
1321 cttggtctctattatattggtccggtggacgggcacaatattgatgatcttattgccatt ctcaaagaggtgaagagtaccaaaacaacaggtcctgtcctaatccatgttgtcaccgag
     L  G  L  Y  Y  I  G  P  V  D  G  H  N  I  D  D  L  I  A  I  L  K  E  V  K  S  T  K  T  T  G  P  V  L  I  H  V  V  T  E
1441 aaaggtcgggggtatccatatgctgagagagctgcggacaagtaccacggagtgaacaag tttgatcccgcaactggaaagcagtccaaggcaagtgctagcacacagtcttacacaaca
     K  G  R  G  Y  P  Y  A  E  R  A  A  D  K  Y  H  G  V  N  K  F  D  P  A  T  G  K  Q  S  K  A  S  A  S  T  Q  S  Y  T  T
1561 tatttgcagaggctttgattgcagaagctgaggcggataaagatgttgttgcaattcatgctgcaatgggaggagggacaggcttaaatctcttccttcgccgttttccgacaagatgt
     Y  F  A  E  A  L  I  A  E  A  E  A  D  K  D  V  V  A  I  H  A  A  M  G  G  G  T  G  L  N  L  F  L  R  R  F  P  T  R  C
1681 tttgatgtggggatcgcagaacagcatgctgttacttttgctgcgggacttgcctgtgaa ggtcttaaacctttttgcgcaatctactcgtctttcttgcagagggcttatgaccaggtg
     F  D  V  G  I  A  E  Q  H  A  V  T  F  A  A  G  L  A  C  E  G  L  K  P  F  C  A  I  Y  S  S  F  L  Q  R  A  Y  D  Q  V
1801 gtacatgatgttgatttgcagaaacttccagtaagattcgcaatggacagagctgggttg gtgggagcagatggtcccacacattgtggagcttttgatgtcacttatatggcatgcctc
     V  H  D  V  D  L  Q  K  L  P  V  R  F  A  M  D  R  A  G  L  V  G  A  D  G  P  T  H  C  G  A  F  D  V  T  Y  M  A  C  L
1921 cctaacatggttgttatggctccttctgatgaggcagagcttttccacatggttgctact gctgctgctatagatgatcgtcctagctgtttccgctacccgagaggtaatggtgttggt
     P  N  M  V  V  M  A  P  S  D  E  A  E  L  F  H  M  V  A  T  A  A  A  I  D  D  R  P  S  C  F  R  Y  P  R  G  N  G  V  G
2041 gttcagttgccaccagaaaacaagggcattcctcttgaggttggaaaaggcaggatattg attgaaggggagagggtggcactcttaggctatggtaccgcagtccagagctgtttagct
     V  Q  L  P  P  E  N  K  G  I  P  L  E  V  G  K  G  R  I  L  I  E  G  E  R  V  A  L  L  G  Y  G  T  A  V  Q  S  C  L  A
2161 gctgcctctttagtggaacgccacggtattcatctgacagttgcagatgccaggttctgc aagccattggacaatgcccttattcgtagccttgcaaaatcacacgaggttttgattaca
     A  A  S  L  V  E  R  H  G  I  H  L  T  V  A  D  A  R  F  C  K  P  L  D  N  A  L  I  R  S  L  A  K  S  H  E  V  L  I  T
2281 gtggaagaaggatcaatcgggggcttcgggtctcatgttgctcatttctggccctggat ggtcttcttgatggcaaactaaagtggcgaccggttgttcttcctgataggtacattgac
     V  E  E  G  S  I  G  G  F  G  S  H  V  A  H  F  L  A  L  D  G  L  L  D  G  K  L  K  W  R  P  V  V  L  P  D  R  Y  I  D
2401 catggatcgccagctgatcagctggtagaagctggtctcacaccatctcacattgcagca acagtattcaatatactcggacaaagaagaaactctctggagattatgtcatcataaaga
     H  G  S  P  A  D  Q  L  V  E  A  G  L  T  P  S  H  I  A  A  T  V  F  N  I  L  G  Q  R  R  N  S  L  E  I  M  S  S  *
2521 agaaggagacaccaatttttgcattcttacaacaacgtacatatagaaggactgtaagatt tatgtgtaaccttcaaatcactgtaaacaatgcagtgtcagttttaggacaacaacatca
2641 gcctgagtcataaacattccagcctgagctatttcagatgctttgtaaggtcatgaatgc aaagccgttgaagcttttggcactgataaaattgaaacctgatagctcttacggcatgtt
2761 tggcatggagccatgaaatgtaataaaatgtgtgatgtaatgatatggtgtcgtgtaaaa gtaacaaaatatgttatgaaatgaaaagtggactggctgattataaatcttcattttggt
2881 ccctgtaatttttgagaaa
```

Supplemental figure 1. Nucleotide and deduced amino acid sequences of *PtDXS* (Potri.008G196500.1). The complete deduced amino acid sequence is depicted in single-letter code beneath the nucleotide sequence. The initiation codon is boxed, an asterisk indicates the termination codon, and conserved functional motifs are underlined.

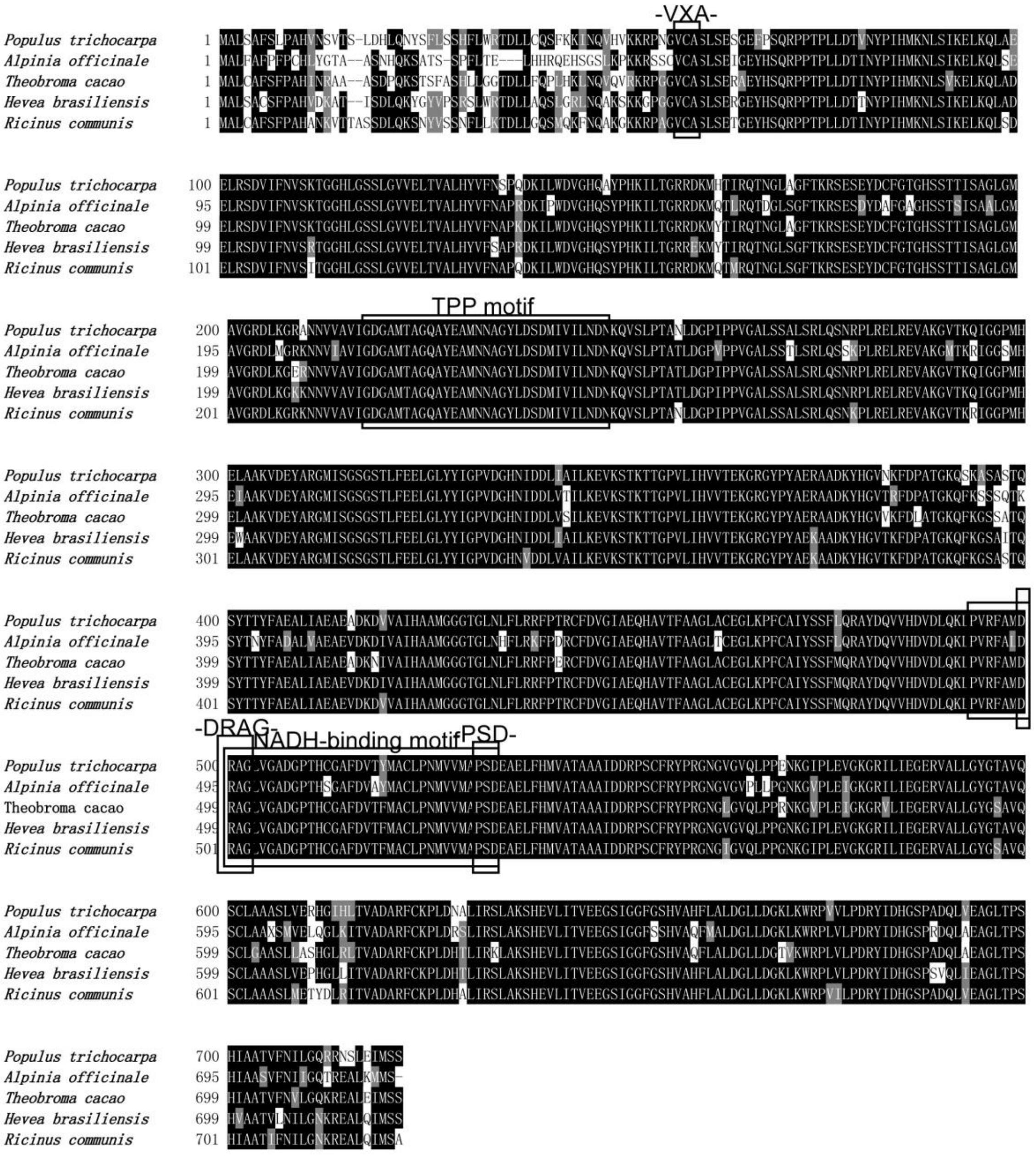

Supplemental figure 2. Deduced amino acid sequences of the conserved regions of *P. trichocarpa PtDXS* and of other *DXS* genes. The TPP motif and DRAG domain are numbered and indicated in the box.

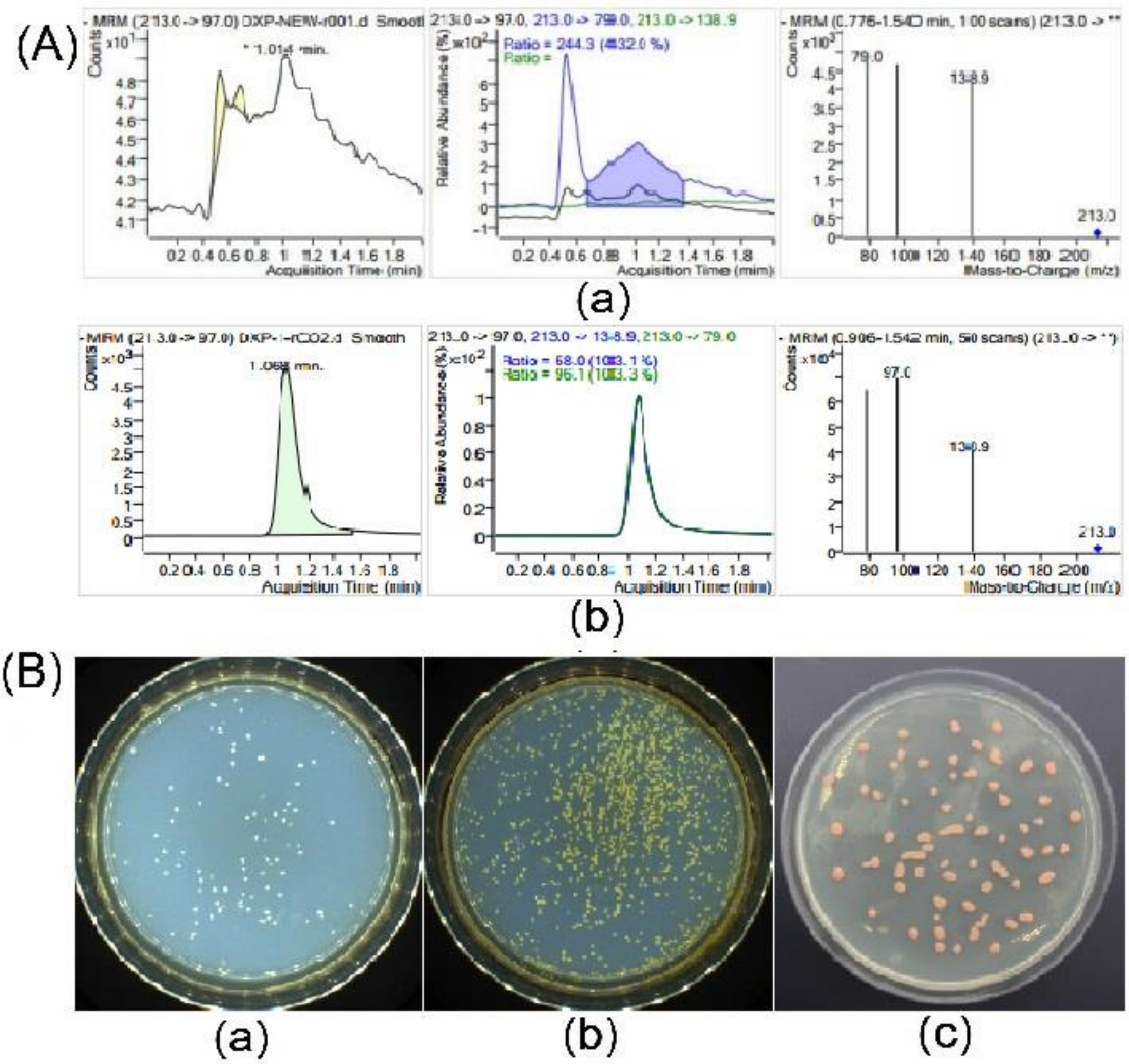

Supplemental figure 3. PtDXS activity in vitro and in vivo. (A) HPLC-MS/MS analysis of DXP (negative control group [a] and sample group [b]). (B) Functional analysis of PtDXS in *E. coli*. Single transformation of pTrc was performed as in [a]. *E. coli* cells harboring pTrc and pAC-BETA were treated as in [b]. *E. coli* cells harboring pTrc-PtDXS and pAC-BETA were treated as in [c].

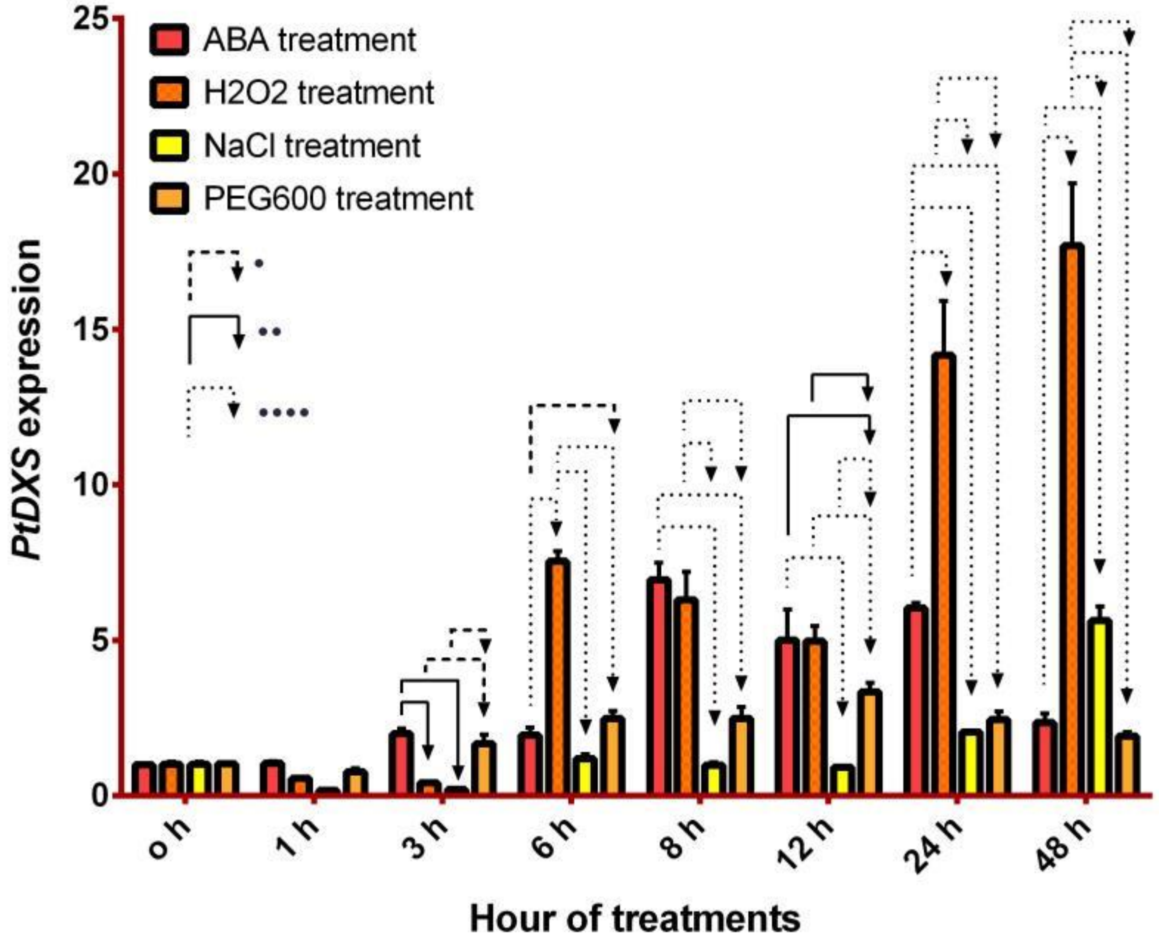

Supplemental Figure 4. Two-way ANOVA has been used to analyze the interaction between different abiotic stresses on *PtDXS* gene expression. *, **, and ****, $P < 0.05$, $P < 0.01$, and $P < 0.0001$, respectively.

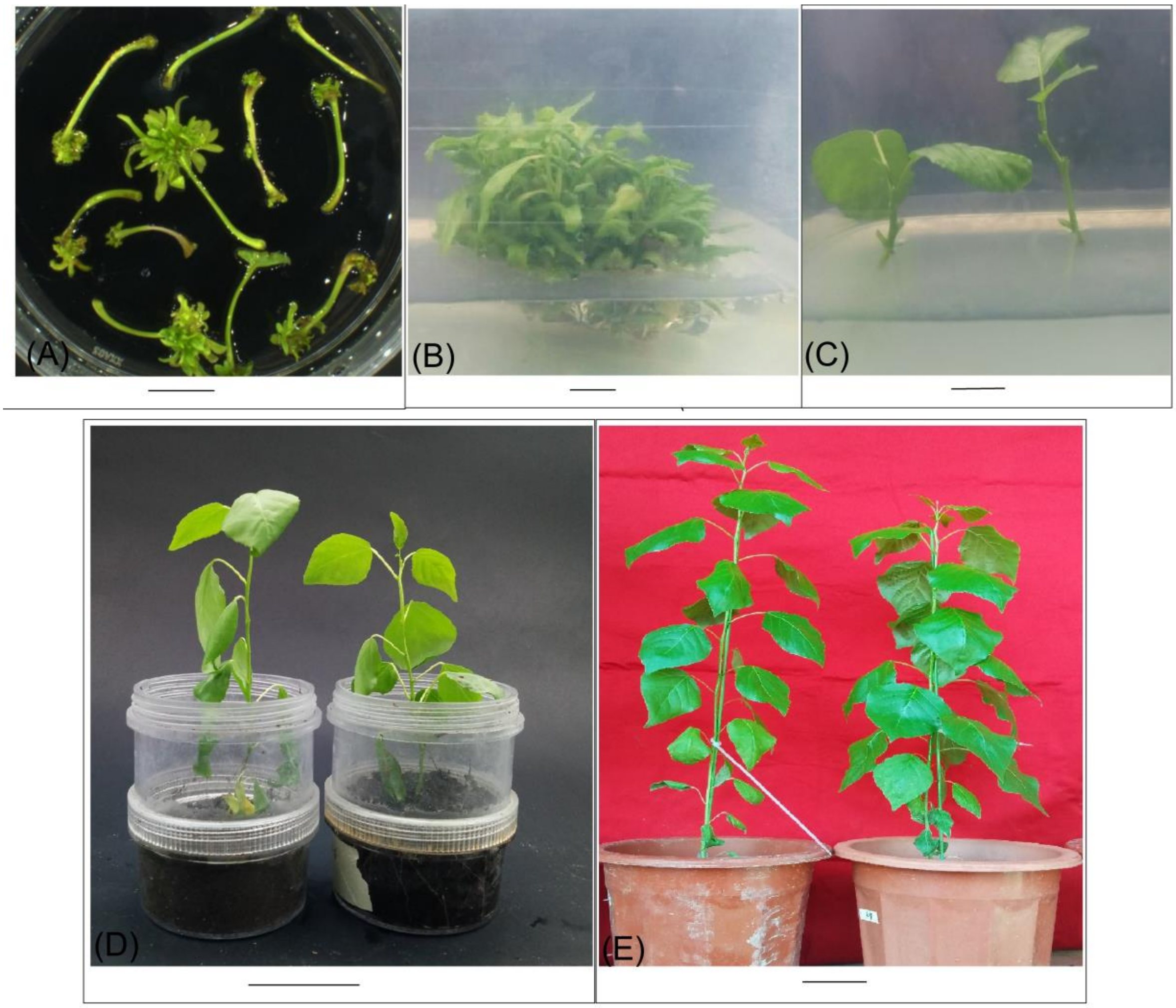

Supplemental figure 5. Regeneration of transformed explants on selective MS medium and regeneration of transgenic poplars in soil. (A) After co-cultivation of leaf pieces and petioles with *Agrobacterium* EHA105 containing *PtDXS*, transformed poplar plants were selected on regeneration medium supplemented with 30 mg/mL kanamycin. (B) Shoots were selected on bud elongation medium supplemented with 20 mg/mL kanamycin. (C) Roots were selected on half-strength MS medium supplemented with 10 mg/mL kanamycin. (D) and (E) Transgenic poplar seedlings grown in soil matrix. A and B Bar=1 cm. C Bar=1.5 cm. D Bar=3 cm. E Bar=6 cm.

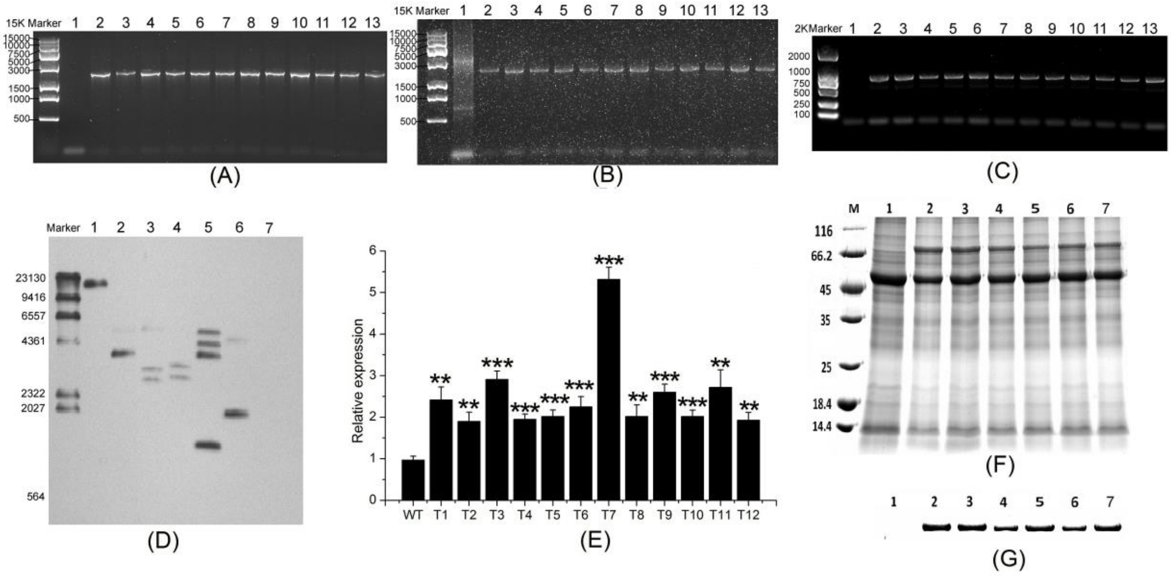

Supplemental figure 6. Identification of regenerated *PtDXS* transgenic poplar plants. (A) Detection of the *PtDXS* gene in transgenic lines and WT through PCR using the genome as template, the primer of the 35S promoter as the upstream primer, and the primer PtDXS-R as the downstream primer. Lane 1: negative control; lanes 2–13: transgenic lines 1–12 (T1-T12), respectively. (B) Detection of the *PtDXS* gene in transgenic lines and WT through PCR using the genome as template, the primer of the PtDXS-F as the upstream primer, and the primer PtDXS-R as the downstream primer. Lane 1: negative control; lanes 2–13: transgenic lines 1–12 (T1-T12), respectively. (C) Detection of the *kanamycin* gene in transgenic lines and WT through PCR using the genome as template, the primer of the Kan-F as the upstream primer, and the primer Kan-R as the downstream primer. Lane 1: negative control; lanes 2–13: transgenic lines 1–12 (T1-T12), respectively. (D) Southern blot analysis of transgenic poplars. Lane M: Marker. Lane 1: The vector PBGWB9-PtDXS digested with EcoRI. Lanes 2-6: Transgenic lines 1-5 (T1-T5) digested with *EcoRI*. Lanes 7: Wild-type poplars digested with *EcoRI*. (E) qRT-PCR analysis of *PtDXS* expression in WT and transgenic plants (T1-T12). (F) SDS-PAGE analysis of PtDXS levels in WT and transgenic plants. Lane M: protein markers; lanes 1–9: WT, T1, T2, T3, T7, T9, and T10 plants, respectively. (G) Western blot of PtDXS protein using a monoclonal antibody against the 6*His tag. Lane M: Marker. Lane 1: Proteins from wild-type poplars. Lanes 2-7: Proteins from transgenic lines (T1, T2, T3, T7, T9, and T10), respectively.

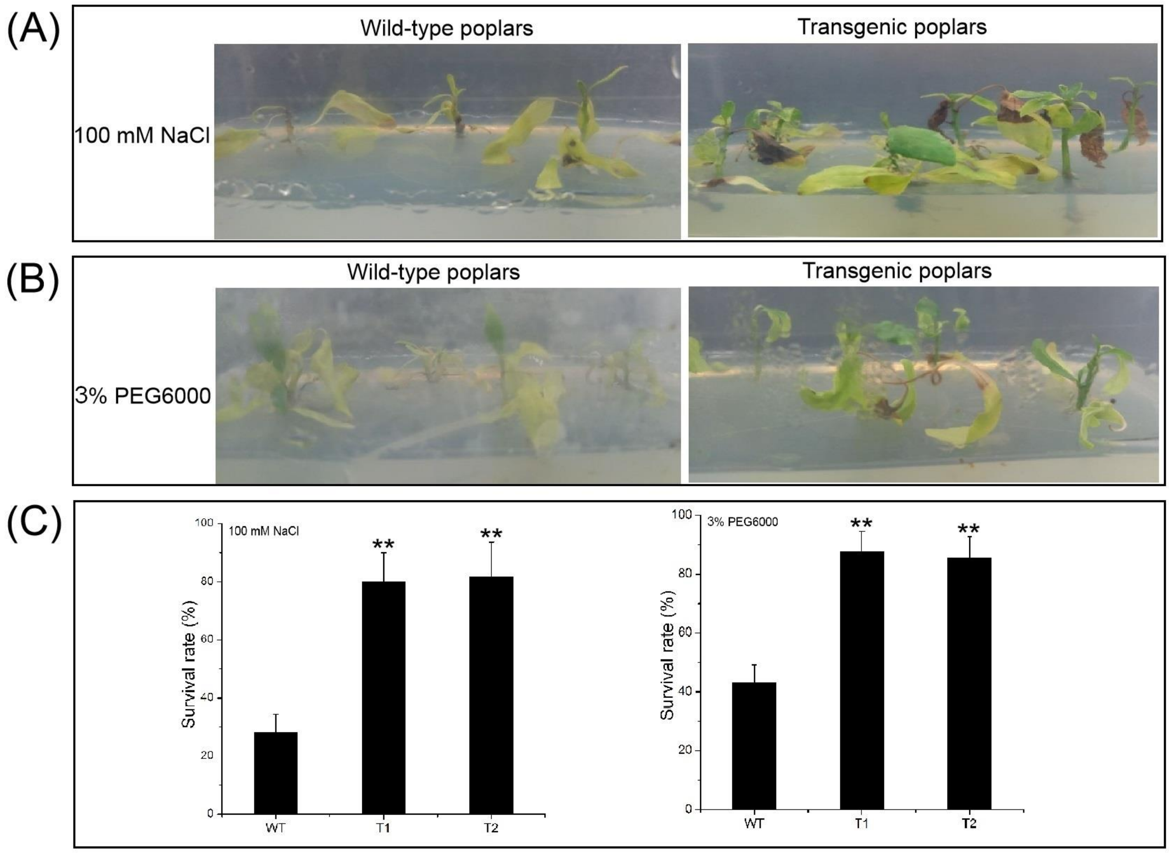

Supplemental figure 7. Rooting abilities of wild-type and transgenic poplars under 100 mM NaCl and 3% PEG6000. (A) Phenotype of WT and transgenic poplars treated with 100 mM NaCl. (B) Phenotype of WT and transgenic poplars treated with 3% PEG6000. (C) Survival rates after 100 mM NaCl and 3% PEG6000 treatment. *, **, and ***, $P < 0.05$, $P < 0.01$, and $P < 0.001$, respectively.

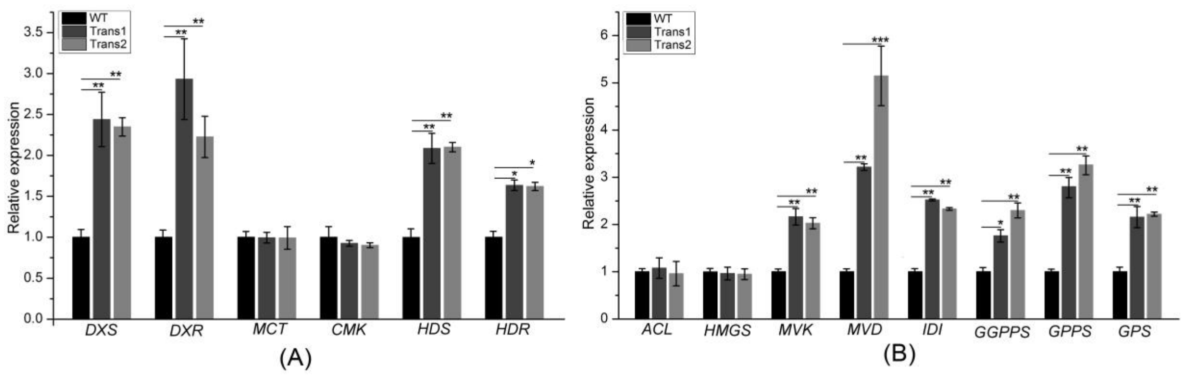

Supplemental figure 8. Expression levels of MEP- and MVA-related genes in the transgenic poplars overexpressing *PtHMGR* and WT poplars (Control). (A) Expression levels of *DXS*, *DXR*, *MCT*, *CMK*, *HDS*, and *HDR*. (B) Expression levels of *ACL*, *HMGS*, *MVK*, *MVD*, *IDI*, *GPS*, *GPPS*, and *GGPPS*. Vertical bars represent means ± SE (n = 3). Three independent experiments were performed. *, **, and ***, P < 0.05, P < 0.01, and P < 0.001, respectively.

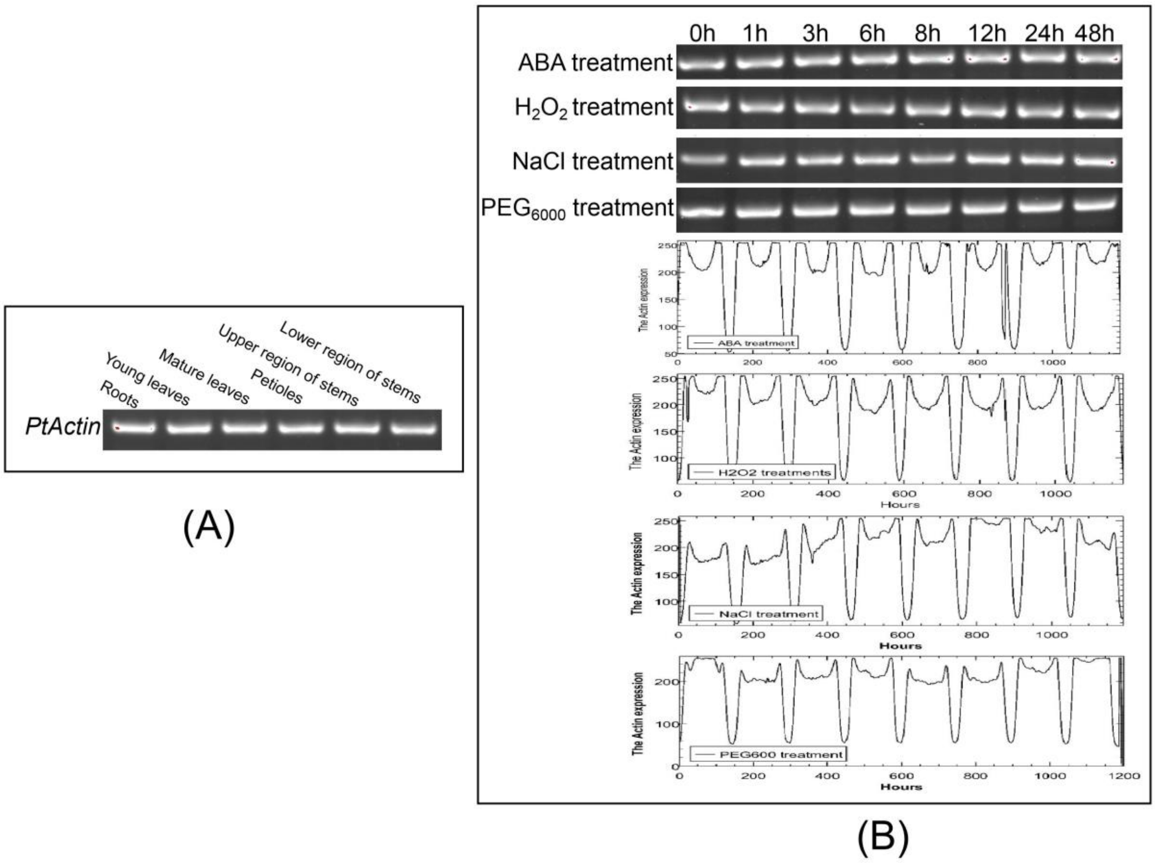

Supplemental figure 9. The *βactin* gene was selected as a reference gene. The *βactin* expression in different organs of poplars and experimental conditions was investigated by RT-PCR. (A) The expression of β*actin* in roots, young and mature leaves, petioles, and upper and lower regions of stems. (B) The expression of *βactin* under the 200 μM ABA, 2 mM H_2O_2, 200 mM NaCl treatment and 10% PEG6000 treatments.
